# PBAF loss leads to DNA damage-induced inflammatory signaling through defective G2/M checkpoint maintenance

**DOI:** 10.1101/gad.349249.121

**Published:** 2022-07-01

**Authors:** Hugang Feng, Karen A. Lane, Theodoros I. Roumeliotis, Penny A. Jeggo, Navita Somaiah, Jyoti S. Choudhary, Jessica A. Downs

**Affiliations:** 1 The Institute of Cancer Research, London SW3 6JB, United Kingdom;; 2 Genome Damage and Stability Centre, University of Sussex, Brighton BN1 9RQ, United Kingdom;; 3 The Royal Marsden National Health Service Foundation Trust, London SM2 5PT, United Kingdom

**Keywords:** SWI/SNF, BAF, PBRM1, BAF180, DREAM complex, p21, TP53, G2/M checkpoint, immunotherapy, cGAS, DNA damage, inflammatory signaling

## Abstract

The PBRM1 subunit of the PBAF (SWI/SNF) chromatin remodeling complex is mutated in ∼40% of clear cell renal cancers. PBRM1 loss has been implicated in responses to immunotherapy in renal cancer, but the mechanism is unclear. DNA damage-induced inflammatory signaling is an important factor determining immunotherapy response. This response is kept in check by the G2/M checkpoint, which prevents progression through mitosis with unrepaired damage. We found that in the absence of PBRM1, p53-dependent p21 up-regulation is delayed after DNA damage, leading to defective transcriptional repression by the DREAM complex and premature entry into mitosis. Consequently, DNA damage-induced inflammatory signaling pathways are activated by cytosolic DNA. Notably, p53 is infrequently mutated in renal cancer, so PBRM1 mutational status is critical to G2/M checkpoint maintenance. Moreover, we found that the ability of PBRM1 deficiency to predict response to immunotherapy correlates with expression of the cytosolic DNA-sensing pathway in clinical samples. These findings have implications for therapeutic responses in renal cancer.

## Abstract (precis)

Here, Feng et al. investigated the molecular mechanisms underlying the responses to immunotherapy in renal cancer by loss of PBRM1, a subunit of the PBAF (SWI/SNF) chromatin remodeling complex mutated in ∼40% of clear cell renal cancers. They found that in the absence of PBRM1, p53-dependent p21 up-regulation is delayed after DNA damage, leading to defective transcriptional repression by the DREAM complex and premature entry into mitosis, and that the ability of PBRM1 deficiency to predict response to immunotherapy correlates with expression of the cytosolic DNA-sensing pathway in clinical samples.

The mammalian family of SWI/SNF chromatin remodeling complexes can be divided into three classes based on subunit composition. These are BAF (BRG1/BRM-associated factors), PBAF (polybromo-associated BAF), and ncBAF or GBAF (noncanonical BAF or GLTSCR1/1L-associated BAF). PBRM1 (or BAF180) is a subunit of PBAF but is not found in either BAF or ncBAF. The PBRM1 gene is frequently mutated in cancer, with loss-of-function mutations particularly prevalent in clear cell renal cell carcinoma (ccRCC).

The development of immune checkpoint inhibitors (ICIs) has had a profound impact on clinical outcomes across a range of cancer types. However, responses are variable, so it is critical to identify predictive biomarkers. Interestingly, PBRM1 mutational status has been identified as a potential predictive biomarker of ICI treatment response, with loss-of-function mutations correlating with improved response (Miao et al. 2018; Braun et al. 2020; Courtet et al. 2020; Dizman et al. 2020). PBRM1 and two other PBAF-specific subunits (ARID2 and BRD7) were identified in a screen for genes regulating sensitivity to T-cell-mediated killing (Pan et al. 2018), and PBRM1 loss results in elevated transcriptional levels of interferon γ-regulated genes (Miao et al. 2018; Pan et al. 2018). However, other studies found either no predictive value or a negative correlation between PBRM1 mutations and ICI response (McDermott et al. 2018; Hakimi et al. 2020; Liu et al. 2020) and found that interferon-regulated gene transcription was lower in cell lines deficient for PBRM1 (Liu et al. 2020). The reason for the different outcomes from these studies is not clear. To identify factors that influence the impact of PBRM1 loss on immunogenicity, it is important to understand in much greater detail the cellular alterations found in PBRM1-deficient cells and how these contribute to ICI response.

There is an important interplay between DNA damage and innate immune signaling, which can sense and respond to the presence of cytosolic DNA (Li and Chen 2018; Lhuillier et al. 2019; Pilger et al. 2021). Recent work highlighted the importance of DNA damage checkpoints, particularly G2/M, in preventing innate immune signaling (Harding et al. 2017; Chen et al. 2020). PBRM1 and the PBAF complex play a role in the cellular response to DNA damage (for review, see Harrod et al. 2020), and this is a potential mechanism by which IFN signaling could be indirectly affected in PBRM1-deficient tumor cells.

p53 is required for the maintenance of the G2/M checkpoint following DNA damage (Bunz et al. 1998). This is carried out through regulation of the DREAM pathway (Sadasivam and DeCaprio 2013; Engeland 2018; Hafner et al. 2019). Briefly, following DNA damage, p53 up-regulates transcription of p21, which in turn inhibits CDK activity. CDK inhibition has an immediate impact on cell cycle progression through regulation of proteins required for mitotic progression but also has a longer-term impact on cell cycle progression through the assembly of the DREAM complex. In the latter role, the reduction of CDK activity allows accumulation of the unphosphorylated forms of p130 and p107, either of which can then assemble with several other proteins, including Lin54 and E2F4-5 subunits, into the DREAM complex. Once assembled, the DREAM complex represses transcription of target genes (Sadasivam and DeCaprio 2013; Engeland 2018). DREAM target genes include those required for cell cycle progression, such as cyclin B1 (CCNB1), CDK1, and PLK1. Repression of these genes is a relatively late response to DNA damage, which is not required for the initiation of the G2/M DNA damage checkpoint, but rather for its maintenance.

PBAF has been implicated in p53-dependent transcriptional activation (Xia et al. 2008; Burrows et al. 2010; Lee et al. 2016; Cai et al. 2019), suggesting a potential role for PBRM1 in mediating G2/M checkpoint responses. Here, we show that PBRM1 is required for maintaining the G2/M DNA damage checkpoint. Cells lacking PBRM1 have delayed p53-dependent p21 up-regulation, leading to belated remodeling of the DREAM complex and failure to efficiently repress genes that promote cell cycle progression. Consequently, PBRM1-deficient cells progress through mitosis with unrepaired DNA damage, leading to micronuclei and up-regulation of innate immune signaling through the cGAS/STING pathway. Moreover, we found that the ability of PBRM1 mutational status to predict response to ICI therapy in clinical samples is correlated with expression of the cytosolic DNA-sensing pathway. These data provide new insights into the cellular impact of PBRM1 deficiency in renal cancer and have important implications for immunotherapy decisions.

## Results

### The PBRM1 subunit of PBAF is required for G2/M DNA damage checkpoint maintenance

**Figure 1.**
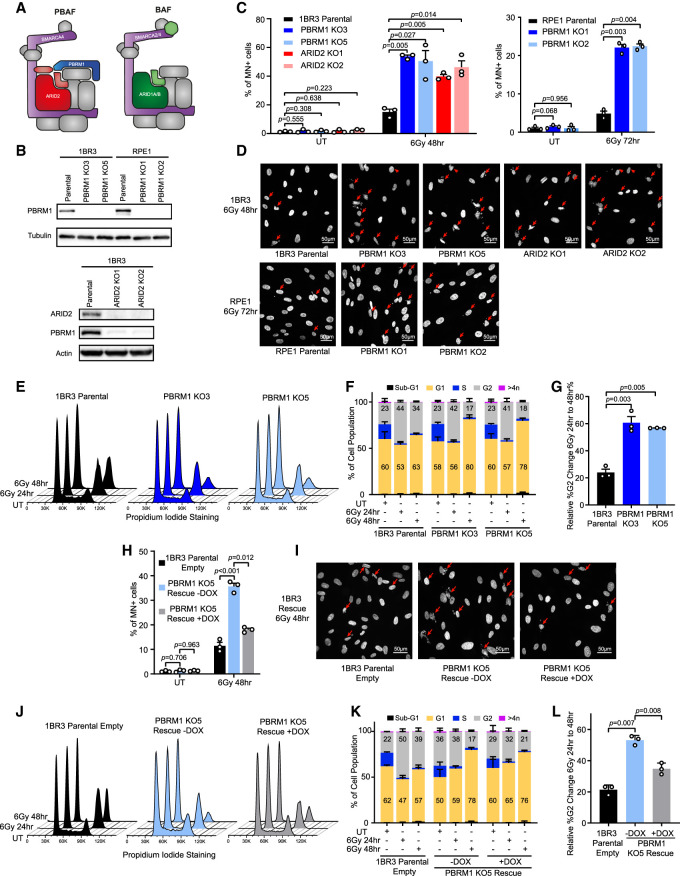
PBRM1 is required for G2/M DNA damage checkpoint maintenance. (*A*) Cartoon illustrating the subunit composition of PBAF and BAF complexes, illustrating core (gray), catalytically active (purple), PBAF-specific (red and blue), and BAF-specific (green) subunits. (*B*) Western blot analysis showing loss of PBRM1 and/or ARID2 expression in the indicated cell lines. (*C*) Quantification of cells with micronuclei in untreated (UT) or irradiated 1BR3 or RPE1 cells with PBRM1 or ARID2 knockout. *n* = 3; mean ± SEM, two-sided paired *t*-test. (*D*) Representative images of DAPI-stained irradiated cells in *C*. Arrows indicate cells with micronuclei. (*E*) Representative FACS profiles of 1BR3 parental and PBRM1 KO cells, untreated (UT) or 24 or 48 h after irradiation. (*F*) Quantification of cell cycle phases from FACS data in *E* with G1 percentage and G2 percentage indicated. *n* = 3; mean ± SEM. (*G*) Quantification of the change in percentage of G2-phase cells at 48 h after irradiation relative to the G2-phase cells at 24 h from FACS data in *F*. *n* = 3; mean ± SEM, two-sided paired *t*-test. (*H*) Quantification of cells with micronuclei in untreated (UT) or irradiated 1BR3 parental cells (1BR3 parental empty) and PBRM1 KO cells with or without re-expression of PBRM1 (PBRM1 KO5 rescue ± DOX). *n* = 3; mean ± SEM, two-sided paired *t*-test. (*I*) Representative images of DAPI-stained irradiated cells in *H*. Arrows indicate cells with micronuclei. (*J*) Representative FACS profiles of 1BR3 parental cells (1BR3 parental empty) and PBRM1 KO cells with or without re-expression of PBRM1 (PBRM1 KO5 rescue ± DOX), untreated (UT) or 24 or 48 h after irradiation. (*K*) Quantification of cell cycle phases from FACS data in *J* with G1 percentage and G2 percentage shown. *n* = 3; mean ± SEM. (*L*) Quantification of the change in percentage of G2-phase cells at 48 h after irradiation relative to the G2-phase cells at 24 h from FACS data in *K*. *n* = 3; mean ± SEM, two-sided paired *t*-test.

To understand more about the contribution of PBAF to DNA damage responses, we created and validated CRISPR–Cas9-generated knockout (KO) clones of the PBAF-specific PBRM1 subunit in two immortalized human cell lines: the fibroblast cell line 1BR3-hTERT and the retinal epithelial cell line RPE1-hTERT (Fig. 1A,B). We additionally generated KO lines in 1BR3-hTERT of another PBAF subunit, ARID2, which results in the loss of stable PBRM1 expression as well (Fig. 1A,B). We tested the response of each of these clonal cell lines to ionizing radiation (IR) by looking at micronuclei as a readout of genome instability. While there was no difference in untreated cells, we found that after treatment with IR, all KO clones in both cell line backgrounds have elevated levels of micronuclei when compared with the isogenic parental cell lines (Fig. 1C,D). To determine whether this was specific to IR-induced DNA damage, we additionally tested the response of these cells to the topoisomerase inhibitor etoposide and found that, similar to IR, there were more micronuclei in the KO clones than in the parental cells (Supplemental Fig. S1A,B).

DNA damage-induced micronuclei arise from cell cycle progression through mitosis with unrepaired DNA damage. We therefore analyzed the G2/M checkpoint response. Following irradiation of asynchronously growing cells, cell cycle distribution was monitored by FACS analysis. We found no difference in the cell cycle profiles of the KO clones compared with the parental cell lines either in untreated conditions or at 24 h after IR (Fig. 1E,F; Supplemental Fig. S1C–F), suggesting that checkpoint initiation is intact in the absence of PBRM1 or ARID2.

Notably, however, there was a greater loss of the arrested G2 population at 48 h following irradiation in the KO lines when compared with the parental lines (Fig. 1D,E; Supplemental Fig. S1C–F). Treatment with an acute dose of etoposide resulted in a G2/M checkpoint response that is maintained over 48 h in the parental cells, but the PBRM1 KO cells show cell cycle progression at 48 h (Supplemental Fig. S1G,H). We quantified the difference in the proportion of G2-phase cells at 48 h relative to 24 h as a measure of G2/M checkpoint maintenance and found a significant increase in cell cycle progression in the KO clones in response to both IR and etoposide (Fig. 1G; Supplemental Fig. S1I–K). Finally, we confirmed this by performing FACS staining with the mitotic marker H3S10ph (Supplemental Fig. S1G,H). These data suggest that the PBRM1 KO cells can initiate the G2/M checkpoint following DNA damage but are unable to maintain it.

To determine whether this activity is specific to the PBAF complex and to validate the phenotypes using an orthogonal approach, we performed siRNA-mediated depletion of either PBRM1 or ARID1A, which is a subunit specific to the BAF complex (Fig. 1A). Consistent with the results using PBRM1 KO cell lines, we found that depletion of PBRM1 leads to increased levels of micronuclei following IR treatment (Supplemental Fig. S2A–C). In contrast, ARID1A depletion did not lead to increased micronucleus numbers in irradiated cells (Supplemental Fig. S2A–C). Moreover, we found that PBRM1 depletion, but not ARID1A depletion, resulted in a change in the proportion of cells in G2 phase at late time points following irradiation (Supplemental Fig. S2D–F). These results suggest that PBAF, but not BAF, is important for mediating G2/M checkpoint responses following DNA damage.

Next, we restored PBRM1 expression in one of the PBRM1 KO cell lines by introducing a doxycycline-inducible expression construct (Supplemental Fig. S2G,H) to test whether the phenotypes are rescued by PBRM1 reintroduction. We treated the cells with IR as before and found that KO cells expressing PBRM1 show a rescue of both IR-induced micronuclei and G2/M checkpoint defects when compared with the same cells without DOX induction (Fig. 1H–L; Supplemental Fig. S2I–L), demonstrating that the effects on IR responses in the PBRM1 KO cells are due to loss of PBRM1. Thus, loss of the PBAF-specific subunits PBRM1 or ARID2 leads to altered cell cycle progression and increased micronuclei following DNA damage when compared with isogenic parental cell lines. This is consistent with a failure to maintain the G2/M checkpoint following DNA damage in PBAF-deficient cells.

### The contribution of PBRM1 to G2/M checkpoint responses is p53-dependent

The FACS profiles of the PBRM1- and ARID2-deficient cells at 24 h following IR treatment suggest that G2/M checkpoint initiation in response to DNA damage is intact, but the cells fail to hold the arrest. To look at this more directly, we analyzed key components of the DNA damage response pathway over time following IR in the 1BR3-hTERT, PBRM1 KO, and rescued PBRM1 KO cells.

**Figure 2.**
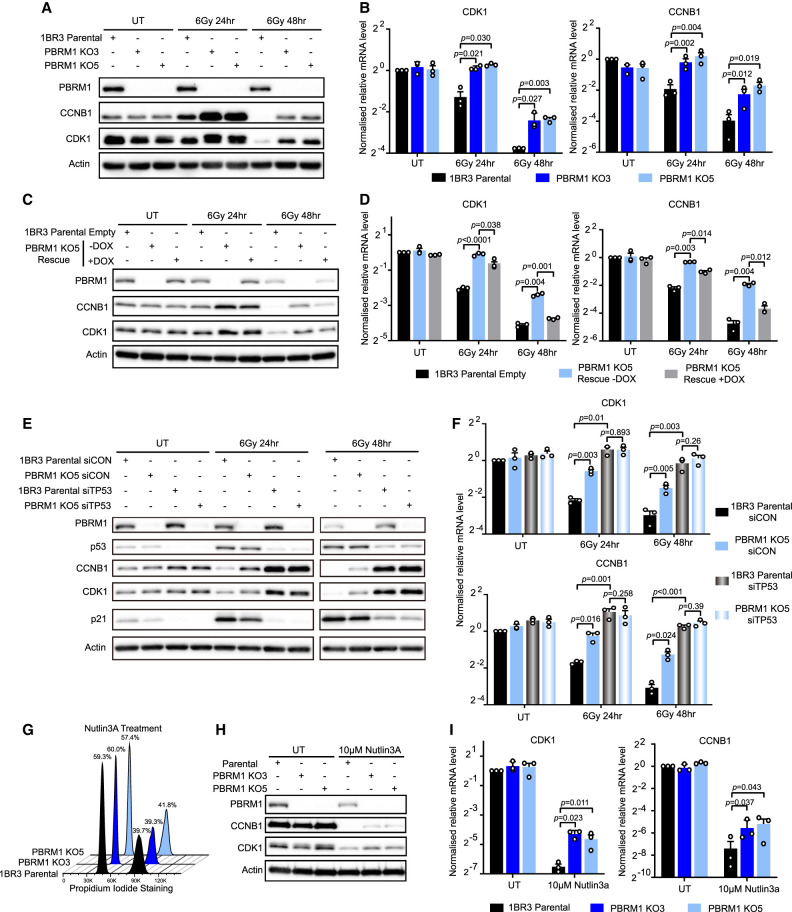
The contribution of PBRM1 to G2/M checkpoint responses is p53-dependent. (*A*) Western blot analysis of cyclin B1 (CCNB1) and CDK1 in untreated (UT) or irradiated 1BR3 parental and PBRM1 KO (KO3/5) cells. (*B*) RT-qPCR analysis of CDK1 or cyclin B1 (CCNB1) expression of cells in *A*. *n* = 3; mean ± SEM, two-sided paired *t*-test. (*C*) Western blot analysis in untreated (UT) or irradiated 1BR3 parental cells and PBRM1 KO cells with or without re-expression of PBRM1 (PBRM1 KO5 rescue ± DOX). (*D*) RT-qPCR analysis of CDK1 or cyclin B1 (CCNB1) expression of cells in *C*. *n* = 3; mean ± SEM, two-sided paired *t*-test. (*E*) Western blot analysis of p53, cyclin B1 (CCNB1), CDK1, and p21 in untreated or irradiated 1BR3 parental and PBRM1 KO5 cells treated with siRNA depletion of TP53 (siTP53) or nontargeting control (siCON). (*F*) RT-qPCR analysis of CDK1 or cyclin B1 (CCNB1) expression of cells in *E*. *n* = 3, mean ± SEM, two-sided paired *t*-test. (*G*) Representative FACS profiles of Nutlin3A-treated 1BR3 parental and PBRM1 KO (KO3/5) cells with quantification. (*H*) Western blot analysis of cyclin B1 (CCNB1) and CDK1 in untreated or Nutlin3A-treated 1BR3 parental and PBRM1 KO (KO3/5) cells. (*I*) RT-qPCR analysis of CDK1 and cyclin B1 (CCNB1) expression of cells in *H*. *n* = 3; mean ± SEM, two-sided paired *t*-test.

Cyclin B1 and CDK1 levels are repressed at late time points in response to DNA damage in a p53-dependent manner, and this is important for G2/M checkpoint maintenance (Engeland 2018). PBAF and, in particular, PBRM1 have been implicated in facilitating p53-dependent transcription (Xia et al. 2008; Burrows et al. 2010; Lee et al. 2016; Cai et al. 2019), raising the possibility that the absence of PBRM1 leads to G2/M checkpoint maintenance defects through defective p53-mediated transcriptional responses. We therefore tested this possibility and found that the PBRM1 KO cell lines have higher levels of both CDK1 and cyclin B1 following IR treatment when compared with the parental cell line (Fig. 2A). We found that the mRNA levels of both target genes are also significantly higher in the irradiated PBRM1 KO cells compared with the irradiated parental cells (Fig. 2B). Moreover, PBRM1 re-expression rescues the damage-induced reduction in both protein (Fig. 2C) and transcript (Fig. 2D) levels, demonstrating that these effects are specific to PBRM1 loss. Interestingly, PBRM1 levels are also reduced at late time points following DNA damage (Fig. 2A,C; Supplemental Fig. S3A,B), raising the possibility of a negative feedback mechanism.

As predicted from the FACS data, we found that the proportions of phosphorylated Chk1 and Chk2 appear similar in all three cell lines (Supplemental Fig. S3C), suggesting that ATR and ATM activation in response to DNA damage are not affected by loss of PBRM1. Consistent with this, we found that RPA focus formation following irradiation was similar in the KO and parental cell lines, suggesting that PBAF loss does not compromise resection (Supplemental Fig. S3D,E). Furthermore, we found no difference in the appearance or resolution of γH2AX foci in these cell lines (Supplemental Fig. S3F,G), indicating that upstream signaling in response to IR is normal in the absence of PBRM1.

We also found that the ARID2 KO cells also have delayed repression of cyclin B1 and CDK1 in response to irradiation (Supplemental Fig. S4A,B), further implicating the PBAF complex in this activity. In addition, CDK1 and cyclin B1 levels were similarly impacted by PBRM1 loss when etoposide was used to induce DNA damage (Supplemental Fig. S4C,D), suggesting that these responses are not limited to irradiation.

Both CDK1 and cyclin B1 are cell cycle-regulated genes, and the cell cycle profile of the KO cells and the parental cells are not equivalent at 48 h after irradiation. This raised the possibility that the changes in CDK1 and cyclin B1 levels were an indirect effect of cell cycle changes and not due to PBRM1-mediated regulation. We therefore used G1 arrest followed by colcemid treatment to determine whether the altered levels of transcripts and proteins observed in the PBRM1 KO cells were a consequence of altered cell cycle profiles (Supplemental Fig. S4G). Under these conditions, the cell cycle profiles of the KO cell lines were similar to those of the parental cells (Supplemental Fig. S4H,I), and we found that there was still a failure to repress expression of CDK1 and cyclin B1 (Supplemental Fig. S4J), indicating that the PBRM1 KO cells have a defect in DNA damage-induced regulation of these genes.

We investigated whether PBRM1 was working in the same pathway as p53. To do this, we depleted p53 and monitored cyclin B1 and CDK1 levels following irradiation in the parental and PBRM1 KO cells. Depletion of p53 in the parental cell line resulted in a failure to repress cyclin B1 and CDK1 protein levels (Fig. 2E) and transcripts (Fig. 2F) following irradiation. There was no further effect of p53 depletion in the PBRM1 KO cells (Fig. 2E,F), suggesting that once p53 is impaired, PBRM1 plays no independent role in mediating the repression of these target genes. In support of this, we also found no increase in micronucleated cells following irradiation in the PBRM1 KO compared with the parental cells when p53 is depleted (Supplemental Fig. S4E,F).

We additionally used Nutlin3A to activate p53 in the absence of DNA damage. In contrast to DNA damage-induced p53 cell cycle arrest, the cells did not re-enter the cell cycle normally following release from Nutlin3A treatment, so we limited analysis of cyclin B1 and CDK1 levels to the 24-h time point when there is no difference in the cell cycle profile of the PBRM1 KO cells when compared with the 1BR3-hTERT parental cells (Fig. 2G). Nutlin3A treatment leads to down-regulation of CDK1 and cyclin B1 in the parental cell line, as expected (Fig. 2H,I). Similar to the results obtained in cells exposed to DNA-damaging agents, we found that CDK1 and cyclin B1 repression was not as pronounced in the PBRM1 KO cell lines at both the protein (Fig. 2H) and mRNA (Fig. 2I) levels in Nutlin3A-treated cells, indicating that PBRM1 is functioning in response to p53 activation. Together, these data show that PBRM1 is working on the same pathway as p53 to mediate DNA damage-induced repression of CDK1 and cyclin B1 to enforce G2/M checkpoint maintenance.

### PBRM1 mediates p53-dependent CDK1 and cyclin B1 repression via the DREAM pathway

**Figure 3.**
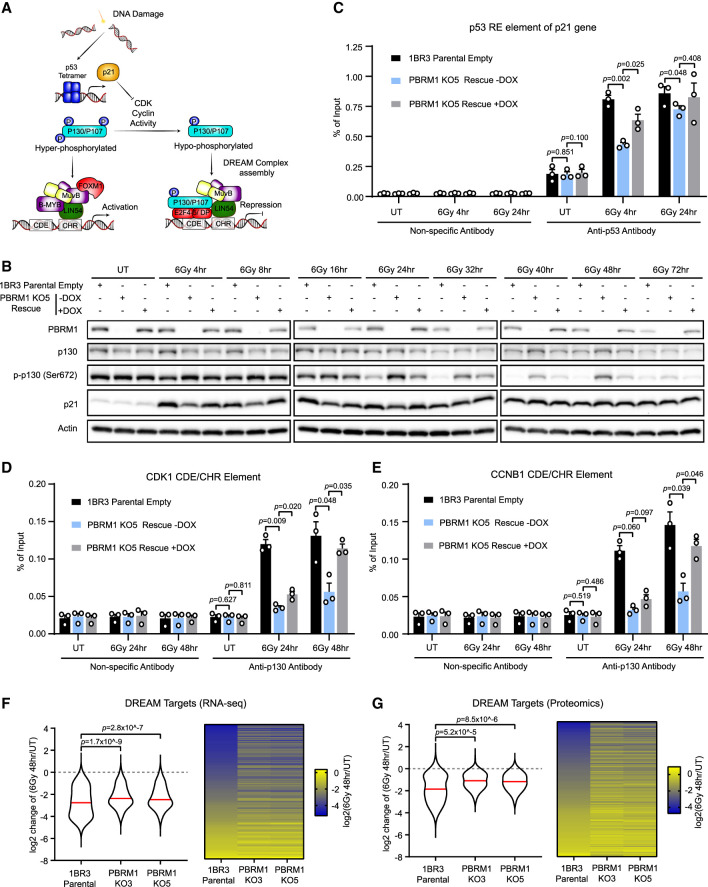
PBRM1 mediates CDK1 and cyclin B1 repression via the DREAM pathway. (*A*) Overview of p53-dependent regulation of the DREAM pathway. p53 up-regulates p21, which inhibits CDK activity, leading to accumulation of the hypophosphorylated forms of p130 and p107, which assemble into the repressive DREAM complex, leading to down-regulation of target genes with CHR/CDE elements including CDK1 and cyclin B1. (*B*) Western blot analysis of total p130 (p130), phospho-p130 (p-p130), and p21 in untreated (UT) or irradiated 1BR3 parental cells and PBRM1 KO cells with or without re-expression of PBRM1 (PBRM1 KO5 rescue ± DOX). (*C*) ChIP-qPCR analysis of p53 binding to the p53 response element (RE) of p21 promoter in untreated (UT) or irradiated 1BR3 parental cells and PBRM1 KO cells with or without re-expression of PBRM1 (PBRM1 KO5 rescue ± DOX), using a nonspecific antibody (negative control) or anti-p53. *n* = 3; mean ± SEM, two-sided paired *t*-test. (*D*,*E*) ChIP-qPCR analysis of p130 binding to the CDE/CHR element of the CDK1 (*D*) or cyclin B1 (*E*) promoter in untreated (UT) or irradiated 1BR3 parental cells or PBRM1 KO cells with or without re-expression of PBRM1 (PBRM1 KO5 rescue ± DOX) using a nonspecific antibody (negative control) or anti-p130. *n* = 3; mean ± SEM, two-sided paired *t*-test. (*F*,*G*) Violin plot and heat map showing changes in DREAM target transcript levels from RNA-seq data (*F*) or DREAM target protein levels from mass spectrometry data (*G*) in 1BR3 parental or PBRM1 KO (KO3 or KO5) cells between untreated (UT) and 48 h after irradiation. Data are plotted as the log_2_ ratio of values from irradiated versus untreated samples [log_2_(6Gy 48 h/UT)]. The red line indicates the median (two-tailed Wilcoxon rank sum test with continuity correction).

CDK1 and cyclin B1 are not directly down-regulated by p53 after DNA damage. Rather, they are indirectly repressed by p53 through regulation of the DREAM pathway (Fig. 3A; Engeland 2018; Hafner et al. 2019). p53 activation leads to transcriptional up-regulation of p21, which then inhibits cyclin/CDK activity. In the absence of damage, cyclin/CDK phosphorylates the p107 and p130 pocket proteins, which prevents them from assembling with Lin54, E2F4-5, and MuvB complex proteins to form the repressive DREAM complex. However, when cyclin B1/CDK1 activity is inhibited by p21 in response to DNA damage, the hypophosphorylated forms of p107 and p130 accumulate, assemble into the DREAM complex, and repress target genes including CDK1 and cyclin B1. This is required to maintain the G2/M checkpoint.

We found that p21 accumulation is defective in the PBRM1 KO line, and this is rescued with re-expression of PBRM1 (Fig. 3B). Notably, however, we found that this defect is mild and only apparent at early time points following irradiation (at 4 and 8 h after IR). p21 levels go up in response to IR even at early time points in the KO cells, and the levels are similar to that in the parental cell line by ∼32 h after IR in the KO cells. These data suggest that p53-mediated transcriptional activation is functional in the absence of PBRM1, but that the response is slower. Consistent with this, we found that p53 accumulation at the p21 promoter is delayed but not abrogated in the PBRM1 KO cell line when p53 binding is monitored by ChIP (Fig. 3C). Re-expression of PBRM1 leads to an increase in p53 recruitment to the p21 promoter (Fig. 3C). These data suggest that PBRM1 facilitates, but is not required for, p53 binding to the p21 promoter.

To determine whether this delay in p21 up-regulation in the KO cells has an impact on DREAM complex assembly and downstream events, we monitored p130 phosphorylation levels over time following irradiation. We found that the hyperphosphorylated form of p130 persists in the PBRM1 KO cell line for much longer after irradiation than in the parental cell line, and this is partially rescued by re-expression of PBRM1 (Fig. 3B). Because only the hypophosphorylated form is competent to assemble into the DREAM complex, the prediction from this finding is that p130 accumulation at DREAM complex target genes is delayed. We interrogated the assembly of p130 into the DREAM complex by performing coimmunoprecipitation assays with the Lin54 subunit. In the parental cells, Lin54 associates with p130 following irradiation, but this is reduced in the PBRM1 KO cells (Supplemental Fig. S5A). Rescue with PBRM1 expression restores this interaction to some extent (Supplemental Fig. S5A).

We additionally monitored p130 binding to the CDK1 and cyclin B1 promoters by ChIP after cells were irradiated. In the parental cell line, p130 accumulation at both promoters is apparent at 24 h after irradiation and increases a bit further by 48 h. In the PBRM1 KO cell line, however, accumulation is substantially reduced at both time points and is rescued by PBRM1 re-expression (Fig. 3D,E). These data show that the inability to efficiently induce p21 after DNA damage leads to defects in DREAM complex assembly at later time points.

We further investigated this pathway in the RPE1-hTERT cell line background. Again, we monitored protein levels following IR treatment and found that the KO cell lines do not repress CDK1 or cyclin B1 as efficiently as the parental cells (Supplemental Fig. S5B). Moreover, phosphorylated p130 persists in the KO cells (Supplemental Fig. S5B). We monitored transcript levels of CDK1, cyclin B1, and two other DREAM targets: cyclin B2 (CCNB2) and PLK1. Consistent with a defect in this pathway, we found that there is a failure to repress all four DREAM targets in the PBRM1 KO clones compared with the parental RPE1 cells (Supplemental Fig. S5C).

Next, we used siRNA in the 1BR-hTERT cell line and found that depletion of PBRM1, but not ARID1A, in the 1BR-hTERT cell line leads to defects in DREAM-mediated repression following DNA damage (Supplemental Fig. S5D,E). These data are again consistent with a role for PBAF, but not BAF, in this process.

The DREAM complex targets a wide range of genes in response to DNA damage. To look at these responses more broadly, we used RNA-seq and mass spectrometry to interrogate the transcriptome and the proteome, respectively, of two PBRM1 KO clones and their isogenic parental cell line 1BR3-hTERT in the absence of IR or 48 h after irradiation. There were 271 annotated DREAM targets in our RNA-seq data set and, as expected, these transcripts were globally reduced in the IR-treated parental cell line compared with the unirradiated cells (Fig. 3F). While the KO cell lines also showed a global reduction in the transcript levels of these genes, the median change in response to IR was significantly less than in the parental cells (Fig. 3F), consistent with a failure to fully repress their transcription in the absence of PBRM1.

Similar trends were apparent in the proteomics data set, where there were 205 annotated DREAM targets detected. We quantified the relative abundance of these proteins and found that DREAM target protein levels in the IR-treated parental cells are lower than in the untreated cells (Fig. 3G). Similar to the transcriptome data, the IR-induced reduction of DREAM target proteins is much less dramatic than in the parental cell line (Fig. 3G). This suggests that the failure to efficiently assemble the DREAM complex in the absence of PBRM1 leads to a global defect in DREAM target repression following DNA damage.

We also analyzed the direct p53 targets in these data sets. As expected, in the parental cells, p53 targets are generally up-regulated in the irradiated data sets compared with the unirradiated (Supplemental Fig. S5F,G). In the PBRM1 KO cell lines, p53 targets are up-regulated to a lesser extent in both the transcriptome and proteome data sets (Supplemental Fig. S5F,G), consistent with PBAF functioning to promote p53-dependent gene expression. However, the differences, while significant, were modest. Since samples were collected 48 h after irradiation, this finding is consistent with our data suggesting that p53-mediated transcriptional responses are delayed but not abrogated in the absence of PBRM1, and it is likely that the differences would be more pronounced at earlier time points.

### PBRM1 influences G2/M checkpoint maintenance in clear cell renal cell carcinoma (ccRCC) cell lines

**Figure 4.**
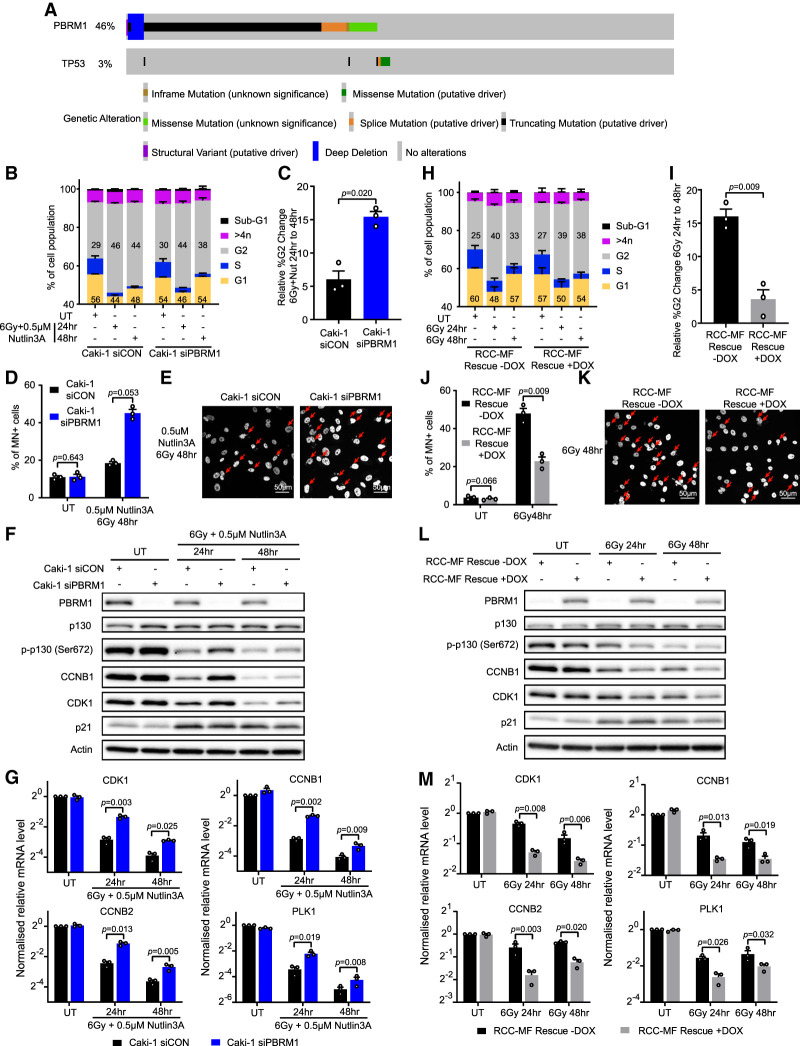
PBRM1 influences G2/M checkpoint maintenance in clear cell renal cell carcinoma cell lines. (*A*) Oncoprint from cBioPortal showing the frequency of genetic alterations of PBRM1 and TP53 in ccRCC samples (TCGA and PanCancer Atlas; *n* = 354). (*B*) Quantification of cell cycle phases from FACS data of untreated (UT) or irradiated and Nutlin3A-treated Caki-1 cells with PBRM1 depletion (siPBRM1) or nontargeting control (siCON). *n* = 3; mean ± SEM. (*C*) Quantification of the percentage change in G2-phase cells at 48 h after irradiation and Nutlin3A relative to G2-phase cells at 24 h after irradiation and Nutlin3A from FACS data in *B*. *n* = 3; mean ± SEM, two-sided paired *t*-test. (*D*) Quantification of cells with micronuclei in untreated (UT) or irradiated and Nutlin3A Caki-1 cells with or without PBRM1 depletion (siPBRM1 or siCON). *n* = 3; mean ± SEM, two-sided paired *t*-test. (*E*) Representative images of DAPI-stained Caki-1 cells in *D*. Arrows indicate cells with micronuclei. (*F*) Western blot analysis in untreated (UT) or irradiated and Nutlin3A Caki-1 cells with or without PBRM1 depletion (siPBRM1 or siCON). (*G*) RT-qPCR analysis of CDK1, cyclin B1 (CCNB1), cyclin B2 (CCNB2), or PLK1 in cells treated as in *F*. *n* = 3; mean ± SEM, two-sided paired *t*-test. (*H*) Quantification of cell cycle phases from FACS data of untreated (UT) or irradiated RCC-MF cells with or without PBRM1 re-expression (rescue ± DOX) with G1 percentage and G2 percentage shown. *n* = 3; mean ± SEM. (*I*) Quantification of the percentage change in G2-phase cells at 48 h after irradiation relative to G2-phase cells at 24 h after irradiation from FACS data in *H*. *n* = 3; mean ± SEM, two-sided paired *t*-test. (*J*) Quantification of cells with micronuclei from untreated (UT) or irradiated RCC-MF cells with or without PBRM1 re-expression (rescue ± DOX). *n* = 3; mean ± SEM, two-sided paired *t*-test. (*K*) Representative images of DAPI-stained irradiated RCC-MF cells in *J*. Arrows indicate cells with micronuclei. (*L*) Western blot in untreated (UT) or irradiated RCC-MF cells with or without PBRM1 re-expression (rescue ± DOX). (*M*) RT-qPCR analysis of CDK1, cyclin B1 (CCNB1), cyclin B2 (CCNB2), or PLK1 expression of cells in *L*. *n* = 3; mean ± SEM, two-sided paired *t*-test.

PBRM1 is frequently mutated in ccRCC, and we wanted to determine whether these activities were likely to play a role in this setting. Because PBRM1 works on the same pathway as p53, PBRM1 loss will not have an impact on G2/M checkpoint maintenance in cells already deficient for p53. Interestingly in this regard, p53 is relatively infrequently mutated in ccRCC, and these mutations rarely co-occur with PBRM1 mutations (Fig. 4A).

We first investigated the role of PBRM1 in G2/M checkpoint responses in the Caki-1 renal cancer cell line, in which both p53 and PBRM1 are intact. While these cells respond to IR with up-regulation of p21, the response to 6 Gy of IR was muted (Supplemental Fig. S6A). We therefore combined irradiation with a low dose of Nutlin3A and found that the cells displayed a more robust p53 response (Supplemental Fig. S6A). Under these conditions, the Caki-1 cells arrested in G2/M, and this was maintained at 48 h after treatment (Fig. 4B; Supplemental Fig. S6B). We then monitored the cells for their ability to maintain the G2/M checkpoint when PBRM1 is depleted. As with non-cancer-derived cell lines, we found that loss of PBRM1 leads to a reduction in the G2/M population at late time points when compared with the nontargeting siRNA (siCON)-treated Caki-1 cells (Fig. 4B,C; Supplemental Fig. S6B). Moreover, the siPBRM1 cells had a greater number of micronuclei than the siCON-treated cells (Fig. 4D,E), suggesting that PBRM1 is important for preventing progression through mitosis with unrepaired DNA damage.

We then interrogated p53-dependent signaling events and found that the siPBRM1 cells show persistent phosphorylated p130 and higher levels of CDK1 and cyclin B1 following treatment with Nutlin3A and IR (Fig. 4F). Additionally, transcription of the DREAM target genes CDK1, cyclin B1, cyclin B2, and PLK1 is less efficiently repressed when PBRM1 is depleted (Fig. 4G), suggesting that the p53-dependent DREAM pathway is also dependent on PBRM1 in the Caki-1 ccRCC cell line.

To explore this further, we used the RCC-MF ccRCC cell line, which is p53-proficient but has a homozygous frameshift mutation in PBRM1 (c1583del). We used the doxycycline-inducible PBRM1 expression construct to create a stable cell line with restored PBRM1 expression (Fig. 4L; Supplemental Fig. S6C). We then treated the cells with IR, monitored cell cycle progression, and found that the PBRM1-expressing cells hold the G2/M checkpoint more efficiently than the PBRM1-deficient cells (Fig. 4H,I; Supplemental Fig. S6D). In addition, after irradiation, there were fewer micronuclei in the RCC-MF cells when PBRM1 is expressed (Fig. 4J,K).

Furthermore, the IR-induced loss of phosphorylated p130 occurred more rapidly when PBRM1 is expressed in the RCC-MF cells (Fig. 4L), and cells expressing PBRM1 had lower levels of CDK1 and cyclin B1 after irradiation than the controls (Fig. 4L). The differences at the protein level were somewhat modest, but the ability to repress transcription in response to IR when PBRM1 expression was restored was more apparent (Fig. 4M). PBRM1 is therefore important for regulation of the p53-dependent DREAM pathway and maintenance of the G2/M checkpoint in response to damage in p53-proficient ccRCC cancer cells.

### PBRM1-deficient cells show up-regulated DNA damage-induced inflammatory signaling

Mitotic progression with unrepaired DNA damage that results in micronucleus formation can lead to innate immune responses at least in part through activation of the cGAS/STING pathway (Li and Chen 2018; Lhuillier et al. 2019; Pilger et al. 2021). cGAS is a DNA binding protein that recognizes cytosolic DNA and activates a downstream signaling pathway that leads to up-regulation of type I interferons and cytokine genes (Li and Chen 2018). Recent work highlighted the importance of G2/M checkpoint responses in preventing this inflammatory signaling (Chen et al. 2020). We hypothesized that G2/M checkpoint defects in the absence of PBRM1 would have important indirect effects on the expression of these genes. We therefore investigated whether the failure to maintain the DNA damage G2/M checkpoint in the absence of PBRM1 has any impact on cGAS/STING signaling.

**Figure 5.**
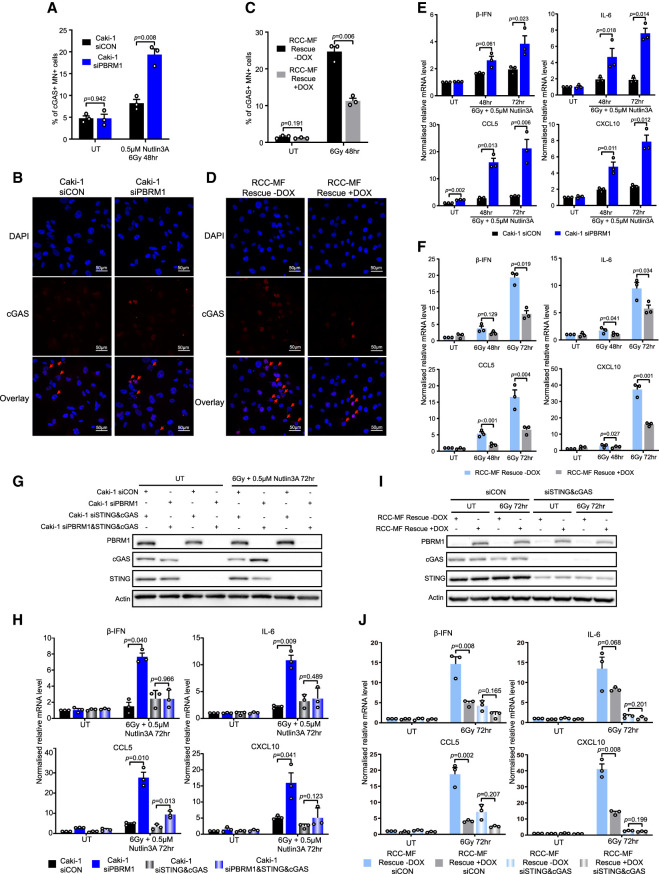
PBRM1-deficient cells show up-regulated DNA damage-induced inflammatory signaling. (*A*) Quantification of cells with cGAS-positive micronuclei in untreated (UT) or irradiated and Nutlin3A-treated Caki-1 cells with PBRM1 depletion (siPBRM1) or nontargeting control (siCON). *n* = 3; mean ± SEM, two-sided paired *t*-test. (*B*) Representative images of irradiated and Nutlin3A-treated Caki-1 cells in *A*. Cells were stained with DAPI and an antibody against cGAS (positive micronuclei are indicated with arrows). (*C*) Quantification of cells with cGAS-positive micronuclei in untreated (UT) or irradiated RCC-MF cells with or without PBRM1 re-expression (rescue ± DOX). *n* = 3; mean ± SEM, two-sided paired *t*-test. (*D*) Representative images of irradiated RCC-MF cells in *C*. Cells were stained with DAPI and an antibody against cGAS. cGAS-positive micronuclei are indicated with arrows. (*E*,*F*) RT-qPCR analysis of b-IFN, IL-6, CCL5, and CXCL10 gene expression in Caki-1 cells with or without PBRM1 depletion (*E*) or RCC-MF cells with or without PBRM1 re-expression (*F*). *n* = 3; mean ± SEM, two-sided paired *t*-test. (*G*) Western blot analysis of cGAS and STING in untreated (UT) or irradiated and Nutlin3A-treated Caki-1 cells with PBRM1 depletion (siPBRM1), STING and cGAS double depletion (siSTING&cGAS), PBRM1, STING, and cGAS triple depletion (siPBRM1&STING&cGAS), or nontargeting control (siCON). (*H*) RT-qPCR analysis of b-IFN, IL-6, CCL5, and CXCL10 expression of Caki-1 cells in *G*. *n* = 3; mean ± SEM, two-sided paired *t*-test. (*I*) Western blot analysis of cGAS and STING in untreated (UT) or irradiated RCC-MF cells with or without PBRM1 re-expression (rescue ± DOX) and with STING and cGAS double depletion (siSTING&cGAS) or nontargeting control (siCON). (*J*) RT-qPCR analysis of b-IFN, IL-6, CCL5, and CXCL10 expression of RCC-MF cells in *I*. *n* = 3; mean ± SEM, two-sided paired *t*-test.

We first tested the cGAS/STING pathway proficiency and found that, to varying degrees, the 1BR3-hTERT, Caki-1, and RCC-MF cell lines are able to sense cytosolic DNA (or polyI:C) (Supplemental Fig. S7A). Using immunofluorescence, we found that there was a greater number of cGAS-positive micronuclei in the PBRM1 KO cells compared with the 1BR3-hTERT parental cells (Supplemental Fig. S7B,C), proportionate to the greater total number of micronuclei in the absence of PBRM1. Similarly, PBRM1-depleted Caki-1 cells treated with IR plus Nutlin3A show a greater number of cGAS-positive micronuclei when compared with the siCON cells (Fig. 5A,B). Finally, re-expression of PBRM1 in RCC-MF cells led to a reduction in cGAS-positive micronuclei compared with the PBRM1-deficient RCC-MF cells following irradiation (Fig. 5C,D).

We used RT-PCR to look at the expression levels of β-IFN (IFNB1), IL-6, CCL5, and CXCL10, which are among the genes up-regulated in a cGAS-dependent manner in response to cytosolic DNA. IL-6 mRNA levels were higher in the PBRM1 KO cells following irradiation when compared with the 1BR3 parental cells (Supplemental Fig. S7D), but we were unable to detect expression of IFNB1, CCL5, or CXCL10 in the 1BR3-hTERT cells either by RT-PCR or RNA-seq, regardless of whether PBRM1 was present or whether the cells had been irradiated. We therefore looked in the ccRCC cell lines, where expression of all four of these genes is detectable. Importantly, DNA damage-dependent up-regulation of all four genes was substantially higher in the siPBRM1 Caki-1 cells (Fig. 5E), suggesting that the increased number of cGAS-positive micronuclei leads to an elevated inflammatory signaling response. Conversely, restored expression of PBRM1 in RCC-MF resulted in substantially lower levels of all four transcripts following IR treatment (Fig. 5F). These data suggest that the ability of PBRM1 to maintain a G2/M checkpoint following DNA damage has important consequences for the magnitude of DNA damage-induced inflammatory signaling.

To determine whether these responses are dependent on the cGAS/STING pathway, we depleted cGAS and STING and repeated the analyses. DNA damage-induced up-regulation of β-IFN, IL-6, CCL5, and CXCL10 was severely reduced when cGAS/STING was depleted in the PBRM1-depleted cells (Fig. 5G,H), suggesting that these responses are being driven primarily by cGAS/STING recognition of cytosolic DNA. Similarly, the up-regulation of these genes in response to DNA damage in the PBRM1-deficient RCC-MF cell line is impaired when cGAS/STING is depleted (Fig. 5I,J). Therefore, the inability of cells to maintain the G2/M checkpoint when PBRM1 is deficient leads to progression through the cell cycle with unrepaired DNA damage, which results in inflammatory signaling driven primarily by cGAS/STING in response to cytosolic DNA.

### Clinical response in patients with PBRM1 LOF mutation is associated with innate immune signaling and cGAS expression

These findings raise the possibility that the PBRM1-dependent DNA damage checkpoint and inflammatory signaling pathway is important for the response to ICI therapy. We therefore probed the data from the three Checkmate clinical trials (009, 010, and 025) (Braun et al. 2020) and the Javelin 101 clinical trial (Motzer et al. 2019) in which ccRCC patients were treated with ICI therapy (nivolumab in Checkmate and avelumab in Javelin) to investigate the relationship between PBRM1 deficiency, cytosolic DNA-sensing pathway regulation, and ICI response. We first identified patients from the Checkmate trials with both RNA-seq and PBRM1 mutational status available and who had also been treated with nivolumab (Supplemental Fig. S8A). We then stratified these by PBRM1 mutational status (WT vs. LOF) and by patient response to ICI therapy (complete or partial response [CR/PR] vs. progressive disease [PD]). Patients with missense mutations of unknown significance, as well as patients with stable disease (SD) or response to ICI not evaluated (NE), were not included in the analysis (Supplemental Fig. S8A). As previously reported (Braun et al. 2020), there is a proportionately better response among patients with PBRM1 LOF (50% with CR/PR in these patients compared with 30% with CR/PR in the PBRM1 WT group).

**Figure 6.**
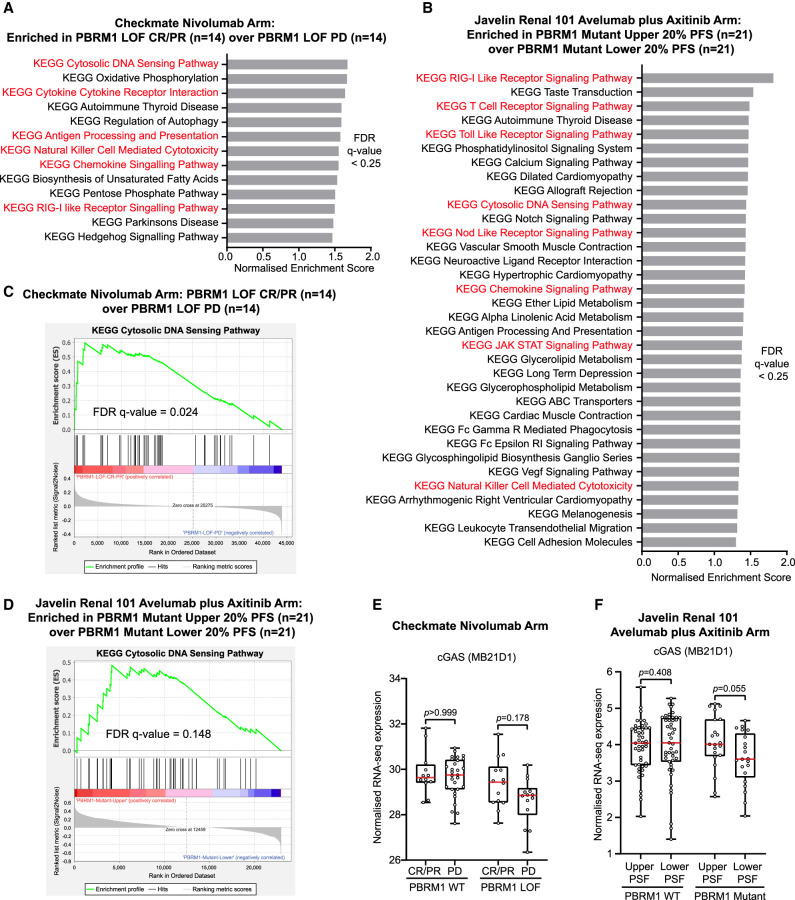
Clinical response in patients with PBRM1 mutation is associated with innate immune signaling and cGAS expression. (*A*,*B*) Significantly enriched KEGG gene sets (false discovery rate [FDR] *q*-value < 0.25) by gene set enrichment analysis (GSEA) from Checkmate trial data in PBRM1 mutant patients treated with nivolumab with complete or partial response (CR/PR) over progressive disease (PD) (*A*) and from Javelin renal 101 trial data in PBRM1 mutant patients treated with avelumab plus axitinib with the upper 20% PFS over the lower 20% PFS (*B*). Immune signaling KEGG gene sets are marked in red. (*C*,*D*) Corresponding gene set enrichment analysis (GSEA) plots of gene set “KEGG cytosolic DNA-sensing pathway” as an example of the analysis in *A* and *B*. The false discovery rate (FDR) *q*-value is indicated. (*E*,*F*) Box and whisker plots showing gene expression of cGAS (MB21D1) from Checkmate trial data in PBRM1 WT or mutant patients treated with nivolumab with complete or partial response (CR/PR) over progressive disease (PD) (*E*) and from Javelin renal 101 trial data in PBRM1 WT or mutant patients treated with avelumab with the upper 20% PFS over the lower 20% PFS (*F*). Each data point represents an individual patient. Box plot hinges denote the 25th–75th percentiles, central lines denote the median, and whiskers extend to the highest and lowest values (Wilcoxon rank sum test).

When all PBRM1 LOF samples are compared with all PBRM1 WT samples regardless of response, no enrichment of pathways related to DNA damage inflammatory signaling is observed (Supplemental Fig. S8B). Strikingly, however, when we compared PBRM1 LOF patient samples that responded well to ICI therapy (CR/PR) with those that responded poorly (PD), we found that the pathway showing the greatest enrichment is the cytosolic DNA-sensing pathway (Fig. 6A), and the correlation is significant (Fig. 6C). Several other immune signaling pathways are also enriched in these samples and correlate with good response (Fig. 6A). In contrast, this pattern of pathway enrichment is not apparent when PBRM1 WT patient samples are similarly analyzed (Supplemental Fig. S8C).

We analyzed the samples from the Javelin trial (Motzer et al. 2019) and found strikingly similar trends. We first identified patients with RNA-seq data and known PBRM1 mutational status that were treated with avelumab, and these were stratified by their response (Supplemental Fig. S9A). We found that patients with PBRM1 mutations who were in the upper 20% of responders according to the length of progression-free survival (PFS) showed enrichment of DNA damage inflammatory signaling pathways, including the cytosolic DNA-sensing pathway, when compared with the lower 20% of responders according to PFS (Fig. 6B,D). Again, this pattern was not observed when comparing all PBRM1 loss-of-function patients with all PBRM1 WT patients (Supplemental Fig. S9B) or when we compared responders and nonresponders in patients without PBRM1 mutations (Supplemental Fig. S9C).

These data suggest that DNA damage inflammatory signaling is associated with ICI response in a subset of PBRM1 LOF patients. Notably, up-regulation of this pathway would depend, at least in part, on the presence of an intact cGAS pathway, and cGAS expression levels are often misregulated in cancer (Yan-Fei et al. 2020). We therefore looked at cGAS expression levels in these samples. When analyzing patients from the Checkmate trials, we found that PBRM1 LOF patients with good response had higher levels of cGAS expression than those with poor response (Fig. 6E). In contrast, in PBRM1 WT patients, cGAS expression levels did not correlate with response to ICI therapy (Fig. 6E). There is a similar trend in PBRM1 mutant samples from the Javelin trial, where patients with longer PFS tended to have higher cGAS expression than patients with shorter PFS. In contrast, there was no clear difference in cGAS expression levels between patients without PBRM1 mutations when they were stratified according to response (Fig. 6F). These data suggest that the levels of cGAS are functionally important and that activation of the cytosolic DNA-sensing pathway influences response to ICI therapy in PBRM1-deficient, but not PBRM1-proficient, patients.

**Figure 7.**
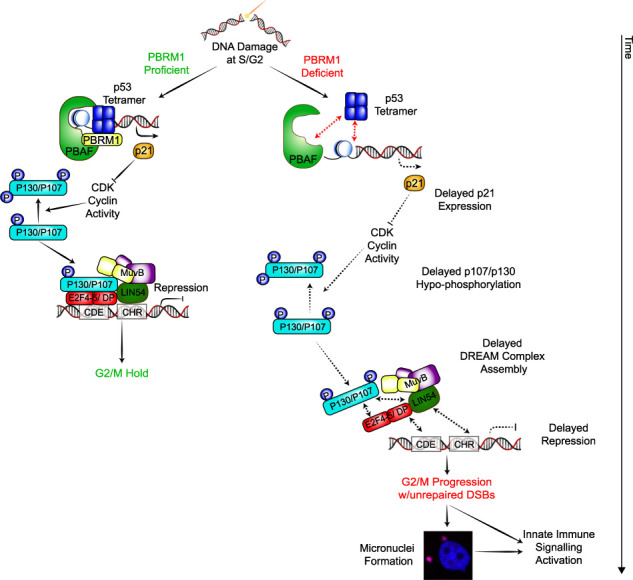
Model for PBRM1-mediated G2/M checkpoint maintenance and its impact on DNA damage inflammatory signaling responses. PBRM1 facilitates p53-dependent transcriptional up-regulation of p21 at early time points following DNA damage. In PBRM1-deficient cells, the delay in p21 up-regulation leads to slower accumulation of hypophosphorylated p130 and, consequently, to delayed repression of DREAM targets, including CDK1 and cyclin B1. This delayed repression in the absence of PBRM1 is sufficient to allow an increase in the number of cells progressing through mitosis with unrepaired damage, leading to increased micronuclei and DNA damage-induced inflammatory signaling.

Taken together, these results support the possibility that the role of PBRM1 in preventing mitotic progression with unrepaired DNA is important for modulating the response to ICI therapy through effects on DNA damage inflammatory signaling (Fig. 7).

## Discussion

Consistent with previous reports, we found that the PBAF chromatin remodeling complex helps to facilitate p53-dependent transcriptional activation (Fig. 7). Specifically, we found a delay in p21 up-regulation within the first 24 h after irradiation that is no longer apparent at later time points. We found that this is specific to PBAF, since depletion of the BAF-specific ARID1A subunit (Fig. 1A) does not have the same effect in these assays. We further show that this relatively mild delay in p53-dependent gene expression when PBRM1 is deficient does not lead to a failure to initiate the G2/M checkpoint, consistent with the idea that a threshold of p21 activity is sufficient to activate immediate downstream signaling. However, the delay in up-regulating p21 leads to more dramatic defects in DREAM complex assembly and function due to the slower accumulation of hypophosphorylated p130. This results in a failure to maintain the G2/M checkpoint after DNA damage and, consequently, re-entry into the cell cycle with unrepaired DNA (Fig. 7). Following this, DNA damage inflammatory signaling is apparent, which is driven by cGAS/STING recognition of cytosolic DNA. These data are consistent with previous reports that showed that mitotic progression with unrepaired damage leads to inflammatory signaling (Harding et al. 2017; Chen et al. 2020) and further highlights the importance of understanding genetic alterations that impair DNA damage-induced checkpoint responses.

We found that PBRM1 and other PBAF subunit transcript levels decrease after DNA damage (Supplemental Fig. S3A,B), suggesting that the PBAF-encoding genes are repressed in response to DNA damage. We considered that this could be a negative feedback loop involving the DREAM pathway. However, the down-regulation is not dependent on PBRM1, and the genes are not among the strictly annotated list of DREAM targets (Engeland 2018). We are currently exploring damage-induced PBAF transcription regulation mechanisms.

Importantly, the ability of PBAF to promote G2/M checkpoint responses is apparent not only in non-cancer-derived cell lines, but also in renal cancer lines. PBRM1 is inactivated in ∼40% of ccRCC cancers, and these are largely mutually exclusive with p53 mutations, suggesting that in renal cancer, PBRM1 is a key determinant of G2/M DNA damage checkpoint responses.

PBRM1 deficiency has been identified in a number of studies as a biomarker for immunotherapy in ccRCC (Miao et al. 2018; Braun et al. 2020; Courtet et al. 2020; Dizman et al. 2020). Evidence suggests that PBAF directly regulates the expression of at least some interferon-responsive genes, but the results are not consistent between studies (Miao et al. 2018; Hakimi et al. 2020). Based on our data, we propose that PBRM1 loss in renal cancer influences ICI responses at least in part through dysregulation of the p53-dependent DREAM pathway. The analysis of clinical samples supports this and suggests that cytosolic DNA sensing and inflammatory signaling is important for response to immunotherapy in patients with PBRM1-deficient tumors.

One prediction from this model is that activation of inflammatory signaling in PBRM1-deficient tumors, and hence their response to immunotherapy, will be most evident when DNA damage is present. Cancer cells often show evidence of replication stress, and this can lead to DNA damage that could contribute to the improved immunotherapy response in patients with PBRM1-deficient tumors. Moreover, long-term hypoxia can lead to down-regulation of DNA repair proteins and increased levels of genome instability, and this is at least in part HIF-independent (Scanlon and Glazer 2015). Angiogenesis inhibitor treatment could therefore result in persistent DNA damage that activates innate immune signaling when PBRM1 is deficient through defective G2/M checkpoint maintenance. In this regard, it is notable that PBRM1 mutations did not correlate with outcome in patients that had no prior therapy (McDermott et al. 2018; Liu et al. 2020), while PBRM1 LOF patients did better when ICI treatment followed treatment with VEGF inhibitors (Miao et al. 2018; Braun et al. 2020), supporting the possibility that prior treatment leads to increased DNA damage-induced inflammatory signaling. However, another study had patients both with and without prior treatment with VEGF inhibitors, and PBRM1 deficiency was not associated with improved outcome in either group (Hakimi et al. 2020), suggesting the involvement of other factors.

The interplay between DNA damage and innate immune signaling is well established. Here, we identify a role for the PBAF chromatin remodeling complex in mediating innate immune signaling indirectly via p53-dependent DNA damage-induced transcriptional repression of G2/M cell cycle genes. Treatments that directly induce DNA damage, such as radiotherapy, are not commonly used with ccRCC, which is considered to be radioresistant. However, it has become increasingly clear that radiation-induced DNA damage can promote antitumor immunity (Li and Chen 2018). The findings presented here raise the possibility that combining DNA damage with ICI therapy in PBRM1-deficient ccRCC could potentiate the therapeutic response.

## Materials and methods

### Cell lines

1BR3-hTERT cells and HEK293T cells were cultured with Dulbecco's modified Eagle medium (DMEM) supplemented with 10% FBS (Gibco) and 1% penicillin/streptomycin (Gibco). RPE1-hTERT cells were cultured with DMEM/nutrient mixture F-12 Ham (Sigma) supplemented with 10% FBS (Gibco), 200 µM glutamax (Gibco), 0.26% sodium bicarbonate (Gibco), and 1% penicillin/streptomycin (Gibco). Caki-1 cells were cultured with McCoy's 5a medium modified (Gibco) supplemented with 10% FBS (Gibco) and 1% penicillin/streptomycin (Gibco). RCC-MF cells were cultured with RPMI 1640 medium supplemented with 10% FBS (Gibco) and 1% penicillin/streptomycin (Gibco). All cell lines were regularly tested for mycoplasma contamination.

### Ionizing radiation and Nutlin3A treatment

Cells were seeded at least 24 h in advance of treatment. Cells were irradiated with the indicated dose by an X-ray source with a 0.6 Gy/min dose rate and the actual dosage was monitored by a Unidose dosimeter. After IR treatment, the culture medium was replaced with fresh (containing Nutlin3A where indicated).

### Plasmids

The CRISPR–Cas9 plasmid pSpCas9(BB)-2A-GFP (PX458) was a gift from Professor Feng Zhang (Addgene 48138). The PBRM1 expression plasmid WT PBRM1-TRIPZ-neo was a gift from Professor William Kaelin (Addgene 107406). The lentiviral packaging plasmids pMD2.G and psPAX2 were gifts from Professor Didier Trono (Addgene 12259 and 12260). The Empty-TRIPZ-neo plasmid was generated by excising the PBRM1 cDNA sequence from the PBRM1-TRIPZ-neo with EcoRI and PacI, and inserting the original fragment from the TRIPZ plasmid (Dharmacon).

### CRISPR–Cas9 knockout (KO) cell line generation

For the generation of PBRM1 and ARID2 CRISPR KO clones, single-guide RNA (sgRNA) sequences for PBRM1 or ARID2 were designed using the Benchling CRISPR sgRNA designing tool (https://www.benchling.com/crispr) and purchased from Sigma (Supplemental Table S2). The sgRNAs were cloned into pSpCas9(BB)-2A-GFP (PX458) according to Ran et al. (2013). Cells were seeded in 10-cm dishes to reach 70% confluence at the time of transfection. For each dish, 10 µg of sgRNA-containing plasmid was transfected into the cells with 20 µL of Lipofectamine 3000 reagent (Invitrogen) and 20 µL of P3000 reagent (Invitrogen) according to the manufacturer's protocol. At 48 h after transfection, the GFP-positive cells were single-cell-sorted using a BD FACSAria III sorter (BD). Clones were screened by Western blot, and putative KO clones were validated with Sanger sequencing and immunofluorescence and are summarized in Supplemental Table S1.

### siRNA-mediated depletion

The PBRM1, ARID1A and nontargeting SMARTpool siRNAs were purchased from Dharmacon Horizon Discovery. Cells were transfected with SMARTpool siRNA at a final concentration of 20 nM in two rounds separated by 24 h using Lipofectamine RNAiMAX transfection reagent (Invitrogen) according to the manufacturer's protocol. Cells were seeded for IR and/or Nutlin3A treatment 24 h after the second transfection.

### Whole-cell extract preparation and Western blot analyses

Cell pellets were lysed in 1× cell lysis buffer (Cell Signaling) with 1× cOmplete EDTA-free protease inhibitor cocktail (Roche) and 1× PhosSTOP phosphatase inhibitor cocktail (Roche) for 30 min on ice and sonicated with Bioruptor Pico (Diagenode). Cell lysate was centrifugated at 13,200 rpm for 15 min at 4°C and the supernatant was collected and quantified. Protein samples were mixed with NuPAGE LDS sample buffer (Life Technology) and 1.25% β-mercaptoethanol (Sigma) and denatured for 5 min at 95°C prior to electrophoresis on either Novex 4%–20% Tris-glycine gel (Thermo Fisher Scientific) or 6% Tris-glycine gel with Precision Plus protein standards (Bio-Rad). Resolved proteins were transferred onto 0.45-µm nitrocellulose membrane (GE Healthcare) and analyzed with the indicated antibodies (Supplemental Table S3).

### Immunofluorescence

Cells were seeded on coverslips at least 24 h prior to treatment. For HEK293T, the coverslips were coated with poly-L-lysine (Sigma). For RPA foci, samples were pre-extracted with 0.2% Triton-phosphate-buffered saline (PBS) for 1 min and then fixed in PBS with 3% paraformaldehyde and 2% sucrose for 10 min at room temperature. For PBRM1, coverslips were fixed in 100% ice-cold methanol for 15 min at −20°C, and for other targets, coverslips were fixed in 4% paraformaldehyde for 10 min at room temperature. The fixed cells were permeabilized with 0.2% Triton-PBS for 3 min. Fixed cells were then blocked with 2% BSA-PBS for 1 h and incubated with primary antibodies (Supplemental Table S3). Coverslips were washed with PBS, incubated with secondary antibody (Supplemental Table S3), and mounted with antifade mounting medium with DAPI (Vector Laboratories). Cells were imaged with an advanced spinning disc confocal microscope using Slidebook 6 software (3i).

γH2AX foci were counted by Cell Profiler software with at least 400 cells analyzed per condition. RPA and Rad51 foci were counted by Cell Profiler software with at least 100 S/G2-phase cells analyzed per condition. Quantification of micronucleated cells was done manually with at least 1000 nuclei (750 nuclei for 1BR3 siRNA) per condition.

### Real-time quantitative PCR (RT-qPCR)

Total RNA extraction was performed with RNeasy mini kit (Qiagen) according to the manufacturer's protocol. One microgram of RNA was reverse-transcribed with SuperScript II reverse transcriptase (Invitrogen) and oligo dT (Invitrogen) according to the manufacturer's protocol. Ten nanograms of cDNA was used as template for each qPCR reaction with 200 nM indicated primers (Supplemental Table S2). qPCR was performed using a StepOnePlus real-time PCR system (Applied Biosystems) on reactions prepared with Power SYBR Green PCR master mix (Applied Biosystems) according to the manufacturer's protocol, with three technical repeats per biological repeat. GAPDH was used for normalization.

### Flow cytometry

Cells were trypsinized, washed with PBS, and fixed in ice-cold 70% ethanol overnight. For propidine iodine (PI) staining, fixed cell pellets were washed in PBS twice and incubated in PBS with 5 µg/mL PI (Invitrogen) and 100 µg/mL RNase (Sigma). Phospho-histone3 antibody (Abcam) was used at a 1:500 dilution in PBS with 0.5% BSA and 0.25% Triton-X for 2 h at room temperature. Cells were washed twice in PBS with 0.25% Triton-X and incubated with goat antimouse/IgG Alexa fluor 647 (Invitrogen) at a 1:500 dilution in PBS with 1% BSA for 30 min in the dark at room temperature. Finally, the cells were stained with PI as described above. Cells were analyzed on the BD LSR II flow cytometer or the BD FACSymphony A5 flow cytometer, with at least 10,000 single-cell events recorded per sample. Quantification and figures were generated with FlowJo v10.6.2 software.

### Chromatin immunoprecipitation (ChIP) with quantitative PCR

Cells were fixed with 1% paraformaldehyde (Electron Microscopy Sciences) for 10 min before quenching with glycine (Sigma). The cell pellets were collected and then incubated in Farnham lysis buffer (5 mM PIPES at pH 8.0, 85 mM KCl, 0.5% NP-40) for 15 min at 4°C. Nucleus pellets were collected by centrifugation at 4°C and incubated in RIPA buffer (1× PBS, 1% NP-40, 0.5% sodium deoxycholate, Roche protease inhibitor cocktail) with 0.3% SDS at a concentration of 3 × 10^7^ cells/mL for 30 min at 4°C. Nuclei were sonicated with Bioruptor Pico (Diagenode) and centrifuged at 13,200 rpm for 15 min at 4°C, and the supernatant containing the fragmented chromatin was collected. The chromatin was diluted and precleared with protein G Dynabeads (Thermo Fisher), and 50 μL was kept as input. For each ChIP, 1 mL of the chromatin (∼1 × 10^7^ cells) was incubated with antibody with 0.5% BSA overnight at 4°C, and then with 50 μL of 0.5% BSA-RIPA-blocked protein G Dynabeads (Thermo Fisher) for 4 h at 4°C. The Dynabeads were washed with RIPA buffer, high-salt wash buffer (100 mM Tris at pH 7.5, 500 mM NaCl, 1% NP-40, 0.5% sodium deoxycholate, 0.1% SDS), LiCl IP wash buffer (100 mM Tris at pH 7.5, 500 mM LiCl, 1% NP-40, 1% sodium deoxycholate), and finally with TE. Immunoprecipitated chromatin was eluted by incubating the Dynabeads with IP elution buffer (1% SDS, 0.1 M NaHCO_3_) for 30 min at 65°C with gentle vortexing. Cross-links were reversed by incubating in 200 mM NaCl, 150 ug/mL RNase A, and 300 ug/mL protease K for 2 h at 37°C and then overnight at 65°C. DNA was purified with QIAquick PCR purification kit (Qiagen).

Quantitative PCR was performed using a StepOnePlus real-time PCR system (Applied Biosystems) with a 15-μL reaction system prepared with Power SYBR Green PCR master mix (Thermo Fisher Scientific 4367659) according to the manufacturer's protocol, with three technical repeats per biological repeat.

### PBRM1 re-expression

Lentivirus was prepared by cotransfecting WT PBRM1-TRIPZ-neo or empty-TRIPZ-neo with psPAX2 and pMD2.G plasmids into HEK293T. Viral particle-containing medium was harvested at 48 and 72 h after transfection, filtered through a 0.45-μm filter, and stored at −80°C. This medium was added to cells to reach a multiplicity of infection of 0.3 with 6 μg/mL polybrene (Sigma). After 24 h, transduced cells were selected with 1.5 mg/mL G418 (Sigma) for 7 d and then maintained in 750 μg/mL G418. The transduced cells were single-cell-sorted using a BD FACSAria III (BD). The clones were screened using immunofluorescence after 1.5 μg/mL doxycycline induction for 72 h and verified by Western blot. Cells were then induced with or without 1.5 μg/mL doxycycline for at least 48 h and seeded 24 h in advance of the indicated treatment in media with or without 1.5 μg/mL doxycycline.

### Coimmunoprecipitation

Cells were lysed in IP lysis buffer (50 mM Tris at pH 7.5, 150 mM NaCl, 0.5% NP-40, Roche protease inhibitor cocktail, Roche phosphatase inhibitor cocktail) supplemented with 3 mM MgCl_2_ and 1 µg/mL benzonase (Thermo Fisher) for 90 min at 4°C. Cell lysate was centrifugated at 13,200 rpm for 15 min at 4°C and the supernatant was collected as total protein (input) and for protein concentration quantification by Bradford assay (Bio-Rad) according to the manufacturer's protocol. For each sample, 75 µL of protein G Dynabeads (Thermo Fisher) was washed with IP lysis buffer and incubated with 2.5 µg of antibody in IP lysis buffer for 2 h at room temperature. Antibody-conjugated Dynabeads were incubated with 2500 µg of total protein in 1 mL of IP lysis buffer overnight at 4°C. Dynabeads were washed four times with cold IP lysis buffer, mixed with 30 µL of NuPAGE LDS sample buffer (Life Technology) and 1.25% β-mercaptoethanol (Sigma), and denatured for 5 min at 95°C. Samples were analyzed by Western blot.

### Proteomic analyses

For sample preparation and TMT labeling, cell pellets were lysed in 150 μL of buffer containing 1% sodium deoxycholate (SDC), 100 mM triethylammonium bicarbonate (TEAB), 10% isopropanol, 50 mM NaCl and Halt protease, and 100× phosphatase inhibitor cocktail (Thermo 78442) on ice, assisted with probe sonication, followed by 5 min at 90°C, and resonicated. Protein concentration was measured with Coomassie Plus Bradford protein assay (Pierce) according to the manufacturer's instructions. Protein aliquots of 50 μg were reduced with 5 mM tris-2-carboxyethyl phosphine (TCEP) for 1 h at 60°C and alkylated with 10 mM iodoacetamide (IAA) for 30 min in the dark, followed by overnight digestion with trypsin at 75 ng/μL (Pierce). Peptides were labeled with the TMT 10plex reagents (Thermo) according to the manufacturer's instructions. The mixture was acidified with 1% formic acid, and the precipitated SDC was removed by centrifugation. Supernatant was dried with a centrifugal vacuum concentrator.

For basic reverse-phase peptide fractionation and LC-MS/MS analysis, peptides were fractionated with high-pH reversed phase (RP) chromatography with the XBridge C18 column (2.1 mm × 150 mm, 3.5 μm; Waters) on a Dionex UltiMate 3000 HPLC system. Mobile phase A was 0.1% (v/v) ammonium hydroxide, and mobile phase B was acetonitrile and 0.1% (v/v) ammonium hydroxide. TMT-labeled peptides were fractionated at a flow rate of 0.2 mL/min using the following gradient: 5 min at 5% B, for 35-min gradient to 35% B, gradient to 80% B in 5 min, isocratic for 5 min, and re-equilibration to 5% B. Fractions were collected every 42 sec, combined in 28 fractions, and vacuum-dried. LC-MS analysis was performed on a Dionex UltiMate 3000 UHPLC system coupled with the Orbitrap Lumos mass spectrometer (Thermo Scientific). Peptides were loaded onto the Acclaim PepMap 100, 100 μm × 2 cm C18, 5-μm trapping column at 10 μL/min flow rate. Peptides were analyzed with the EASY-Spray C18 capillary column (75 μm × 50 cm, 2 μm) at 50°C. Mobile phase A was 0.1% formic acid, and mobile phase B was 80% acetonitrile and 0.1% formic acid. The gradient method included 90-min gradient 5%–38% B, 10 min up to 95% B, 5-min isocratic at 95% B, re-equilibration to 5% B in 5 min, and 10-min isocratic at 5% B at flow rate 300 nL/min. Survey scans were acquired in the range of 375–1500 *m/z* with mass resolution of 120,000, AGC 4 × 105, and maximum injection time (IT) 50 msec. Precursors were selected with the top speed mode in cycles of 3 sec and isolated for CID fragmentation with quadrupole isolation width 0.7 Th. Collision energy was 35% with AGC 1× 104 and maximum IT 50 msec. Quantification was obtained at the MS3 level with HCD fragmentation of the five most abundant CID fragments isolated with synchronous precursor selection (SPS). Quadrupole isolation width was 0.7 Th, collision energy was 65%, and AGC setting 1 × 105 with 105-msec maximum IT. The HCD MS3 spectra were acquired for the mass range 100–500 with 50,000 resolution. Targeted precursors were dynamically excluded for further fragmentation for 45 sec with 7-ppm mass tolerance.

For database search and protein quantification, the mass spectra were analyzed in Proteome Discoverer 2.4 (Thermo Scientific) with the SequestHT search engine. Precursor and fragment ion mass tolerances were set at 20 ppm and 0.5 Da, respectively. Spectra were searched for fully tryptic peptides with maximum two miscleavages. TMT6plex at N terminus/K and carbamidomethyl at C were selected as static modifications. Oxidation of M and deamidation of N/Q were selected as dynamic modifications. Peptide confidence was estimated with the Percolator node, and peptides were filtered at *q*-value < 0.01 based on a decoy database search. All spectra were searched against reviewed UniProt human protein entries. The reporter ion quantifier node included a TMT 10plex quantification method with an integration window tolerance of 15 ppm at the MS3 level. Only unique peptides were used for quantification, considering protein groups for peptide uniqueness. Only peptides with average reporter signal to noise of >3 were used for protein quantification.

### RNA-seq analyses

The RNA-seq library construction, sequencing, and standard data processing were carried out by the Institute of Cancer Research Genomics Facility. Briefly, total RNA extraction was done with Trizol reagent (Invitrogen) and Direct-zol RNA microprep kits (Zymo), and RNA samples were treated with Turbo DNA-free kit (Invitrogen) to remove residual genomic DNA according to the manufacturer's protocol. The total RNA libraries were constructed using the NEBNext rRNA depletion kit (human/mouse/rat) and NEBNext Ultra II directional RNA library preparation kit for Illumina (NEB) according to the manufacturer's protocol. The libraries were sequenced with 100 million 100-bp paired-end reads on a NovaSeq 6000 platform (Illumina). Bcl2fastq software (v2.2.20, Illumina) was used for converting the raw basecalls to Fastqs and to further demultiplex the sequencing data. The STAR alignment software (v.2.5.1b) was used to align reads to the reference genome (GRCh38). HTSeq-count (HTSeq v0.6.1) was used to count the number of reads mapping unambiguously to genomic features. Normalized count data were generated in R using the Bioconductor package DESeq2 (v1.14.1). The normalized data were filtered to remove all rows where the row sum was <1.

### Clinical trial data analysis

The Checkmate 009, 010, and 025 clinical trial data were downloaded from the supplemental material of Braun et al. (2020). The Javelin renal 101 clinical trial data were downloaded from the supplemental material of Motzer et al. (2020). The normalized RNA-seq transcript data were analyzed with gene set enrichment analysis (GSEA v4.1.0) (Subramanian et al. 2005) to measure enrichment of KEGG gene sets (curated gene sets–canonical pathways–KEGG gene sets) between different patient groups with 1000 permutations. The normalized RNA-seq expression level of MB21D1 (cGAS) was extracted and plotted for different patient groups.

### Quantification and statistics analysis

Statistical details of experiments, including number of biological replicates, are included in the figure legends. *P*-values for Student's *t*-test are indicated in the figures. *P*-values for Wilcoxon rank sum test are indicated in the figures.

### Data availability

Source data files have been deposited in Mendeley Data (doi: 10.17632/xkf5fvw6cb.1). The total RNA-seq data reported here has been deposited in the GEO database (accession no. GSE183688). The whole-proteomic analysis data reported here has been deposited in the PRIDE database (accession no. PXD028336).

## Supplementary Material

Supplemental Material
