# Supplementary material for: PBAF loss leads to DNA damage-induced inflammatory signaling through defective G2/M checkpoint maintenance
Source: Genes Dev. 2022 Jul 1;36(13-14):790–806. doi: 10.1101/gad.349249.121 (PMC9480851; doi:10.1101/gad.349249.121)
Supplement: Supplemental Material [file supp_36_13-14_790__DC1.html]

PBAF loss leads to DNA damage-induced inflammatory signaling through defective G2/M checkpoint maintenance — PBAF loss leads to DNA damage-induced inflammatory signaling through defective G2/M checkpoint maintenance — Supplemental Material 

# PBAF loss leads to DNA damage-induced inflammatory signaling through defective G2/M checkpoint maintenance

## Supplemental Material

- Supplemental\_Figure\_S1.pdf
- Supplemental\_Figure\_S2.pdf
- Supplemental\_Figure\_S3.pdf
- Supplemental\_Figure\_S4.pdf
- Supplemental\_Figure\_S5.pdf
- Supplemental\_Figure\_S6.pdf
- Supplemental\_Figure\_S7.pdf
- Supplemental\_Figure\_S8.pdf
- Supplemental\_Figure\_S9.pdf
- Supplemental\_Tabel\_S1.pdf
- Supplemental\_Table\_S2.pdf
- Supplemental\_Table\_S3.pdf
