# Supplementary material for: PBAF loss leads to DNA damage-induced inflammatory signaling through defective G2/M checkpoint maintenance
Source: Genes Dev. 2022 Jul 1;36(13-14):790–806. doi: 10.1101/gad.349249.121 (PMC9480851; doi:10.1101/gad.349249.121)
Supplement: Supplemental Material [file supp_gad.349249.121_Supplemental_Figure_S1.pdf]

Feng_Supplemental Figure S1

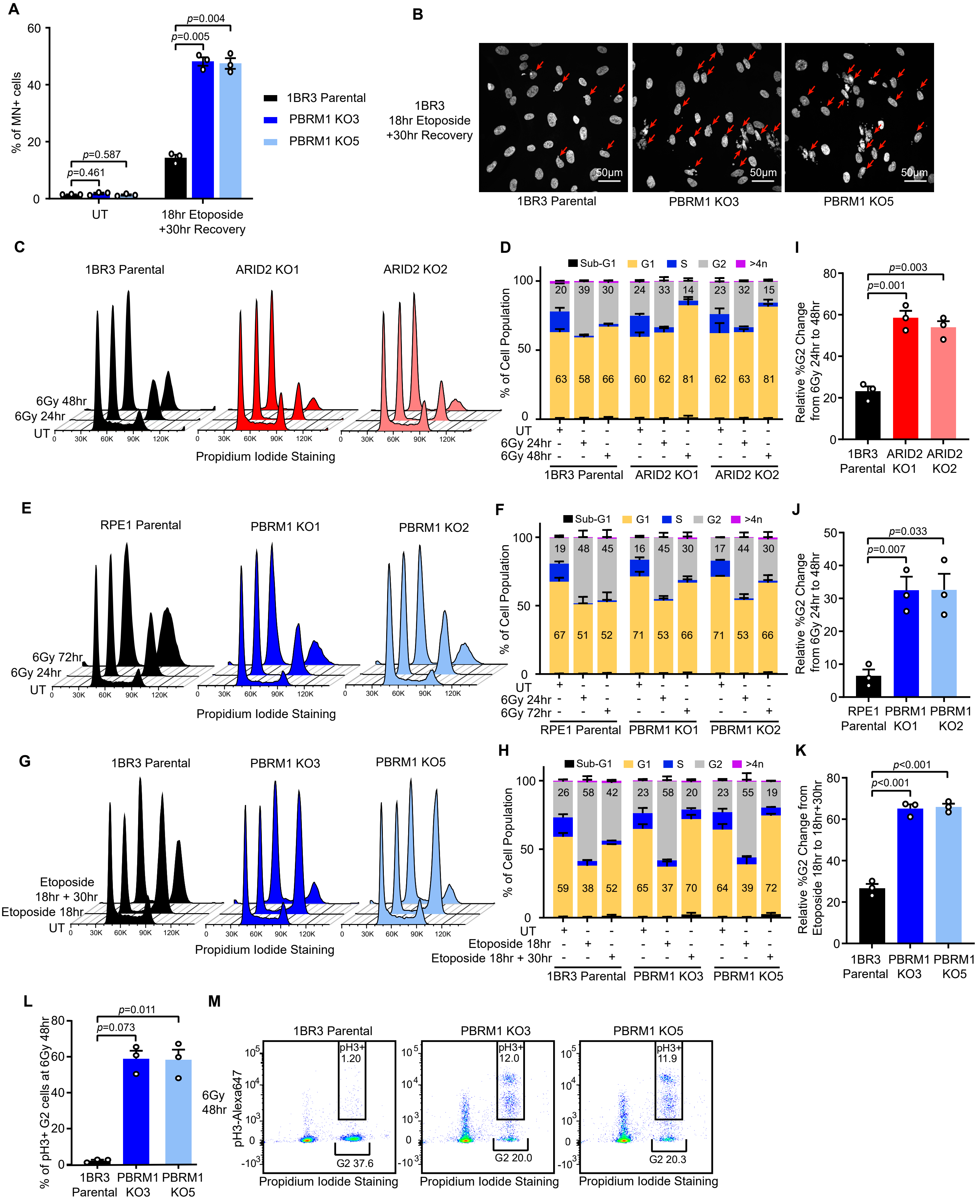

Figure S1. The PBRM1 subunit of PBAF is require for G2/M DNA damage checkpoint maintenance. Related to Figure 1A-F.

(A) Quantification of cells with micronuclei in untreated (UT) or etoposide treated (18h 500nM etoposide treatment with 30h recovery) 1BR3 parental and PBRM1 KO (KO3/5) cells. (n=3, mean±SEM, two-sided paired t test).

(B) Representative images of DAPI stained irradiated cells in (A). Arrows indicate cells with micronuclei.

(C) Representative FACS profiles of 1BR3 parental and ARID2 KO (KO1/2) cells untreated (UT) or 24h or 48h post-irradiation.

(D) Quantification of cell cycle phases FACS data of cells in (C) with G1% and G2%. (n=3, mean±SEM).

(E) Representative FACS profiles of RPE1 parental and PBRM1 KO (KO1/2) cells untreated (UT) or 24 or 72h post-irradiation.

(F) Quantification of cell cycle phases FACS data of cells in (E) with G1% and G2%. (n=3, mean±SEM).

(G) Representative FACS profiles of untreated (UT) or 18h 500nM etoposide treated or 18hr 500nM etoposide treated plus 30hr recovery 1BR3 parental and PBRM1 KO (KO3/5) cells.

(H) Quantification of cell cycle phases from FACS data of cells in (G) with G1% and G2%. (n=3, mean±SEM).

(I) Quantification of the percentage change in G2 phase cells at 48h post-irradiation relative to G2 phase cells at 24h post-irradiation FACS data of cells in (C). (n=3, mean±SEM, two-sided paired t test).

(J) Quantification of the percentage change in G2 phase cells at 72h post-irradiation relative to G2 phase cells at 24h post-irradiation FACS data of cells in (E). (n=3, mean±SEM, two-sided paired t test).

(K) Quantification of the percentage change in G2 phase cells with 18h 500nM etoposide treatment plus 30h recovery relative to G2 phase cells with 18h 500nM etoposide treatment FACS data of cells in (G). (n=3, mean±SEM, two-sided paired t test).

(L) Quantification of H3S10ph positive (pH3+) G2 phase 1BR3 parental and PBRM1 KO (KO3/5) cells at 48h post-irradiation. (n=3, mean±SEM, two-sided paired t test).

(M) Representative FACS profiles of cells in (L) stained with an antibody against H3S10ph (pH3) and propidium iodide.
