# Supplementary material for: PBAF loss leads to DNA damage-induced inflammatory signaling through defective G2/M checkpoint maintenance
Source: Genes Dev. 2022 Jul 1;36(13-14):790–806. doi: 10.1101/gad.349249.121 (PMC9480851; doi:10.1101/gad.349249.121)
Supplement: Supplemental Material [file supp_gad.349249.121_Supplemental_Figure_S2.pdf]

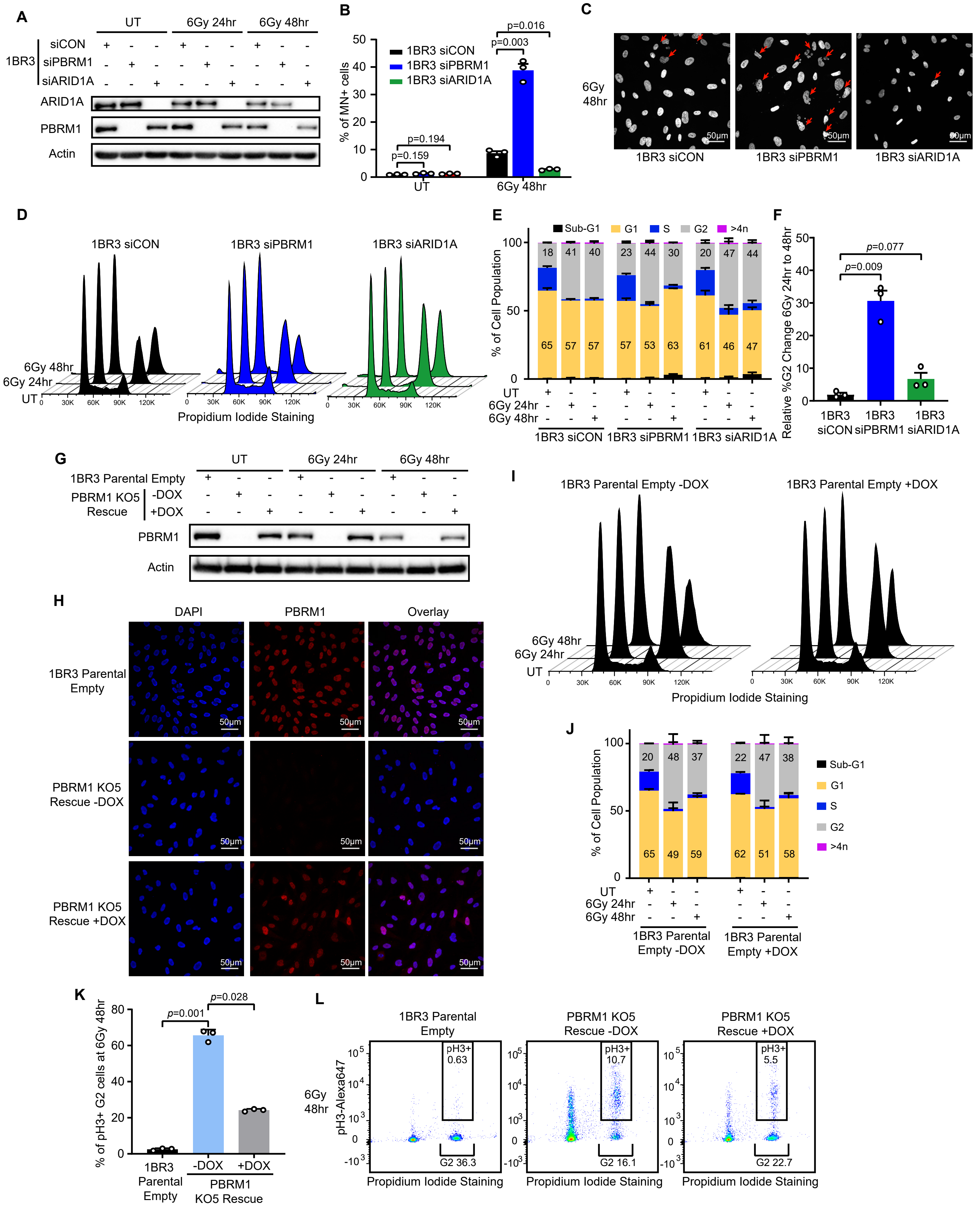
A
UT
6Gy 24hr
6Gy 48hr
1BR3
siCON
siPBRM1
siARID1A
ARID1A
PBRM1
Actin
B
% of MN+ cells
1BR3 siCON
1BR3 siPBRM1
1BR3 siARID1A
p=0.159
p=0.194
p=0.003
p=0.016
UT
6Gy 48hr
C
6Gy 48hr
1BR3 siCON
1BR3 siPBRM1
1BR3 siARID1A
50µm
D
1BR3 siCON
1BR3 siPBRM1
1BR3 siARID1A
6Gy 48hr
6Gy 24hr
UT
Propidium Iodide Staining
E
Sub-G1
G1
S
G2
>4n
% of Cell Population
18 41 40 23 44 30 20 47 44
65 57 57 57 53 63 61 46 47
UT
6Gy 24hr
6Gy 48hr
1BR3 siCON
1BR3 siPBRM1
1BR3 siARID1A
F
Relative %G2 Change 6Gy 24hr to 48hr
p=0.009
p=0.077
1BR3 siCON
1BR3 siPBRM1
1BR3 siARID1A
G
UT
6Gy 24hr
6Gy 48hr
1BR3 Parental Empty
PBRM1 KO5 Rescue
-DOX
+DOX
PBRM1
Actin
H
DAPI
PBRM1
Overlay
1BR3 Parental Empty
PBRM1 KO5 Rescue -DOX
PBRM1 KO5 Rescue +DOX
50µm
I
1BR3 Parental Empty -DOX
1BR3 Parental Empty +DOX
6Gy 48hr
6Gy 24hr
UT
Propidium Iodide Staining
J
% of Cell Population
20 48 37 22 47 38
65 49 59 62 51 58
Sub-G1
G1
S
G2
>4n
UT
6Gy 24hr
6Gy 48hr
1BR3 Parental Empty -DOX
1BR3 Parental Empty +DOX
K
% of pH3+ G2 cells at 6Gy 48hr
p=0.001
p=0.028
1BR3 Parental Empty
-DOX
+DOX
PBRM1 KO5 Rescue
L
1BR3 Parental Empty
PBRM1 KO5 Rescue -DOX
PBRM1 KO5 Rescue +DOX
6Gy 48hr
pH3-Alexa647
pH3+ 0.63
pH3+ 10.7
pH3+ 5.5
G2 36.3
G2 16.1
G2 22.7
Propidium Iodide Staining

Figure S2. The PBRM1 subunit of PBAF is require for G2/M DNA damage checkpoint maintenance. Related to Figure 1G-K.

(A) Western blots showing siRNA depletion of PBRM1 (siPBRM1) or ARID1A (siARID1A) or non-targeting control (siCON) in 1BR3 parental cells.

(B) Quantification of cells with micronuclei in untreated (UT) or irradiated 1BR3 parental following siRNA depletion of PBRM1 (siPBRM1) or ARID1A (siARID1A) or non-targeting control (siCON). (n=3, mean±SEM, two-sided paired t test).

(C) Representative images of DAPI stained irradiated cells in (B). Arrows indicate cells with micronuclei.

(D) Representative FACS profiles of 1BR3 parental cells with siRNA depletion of PBRM1 (siPBRM1) or ARID1A (siARID1A) or non-targeting control (siCON), untreated (UT) or 24h or 48h post-irradiation.

(E) Quantification of cell cycle phases FACS data of cells in (D) with G1% and G2%. (n=3, mean±SEM).

(F) Quantification of the percentage change in G2 phase cells at 48h post-irradiation relative to G2 phase cells at 24h post-irradiation FACS data of cells in (D). (n=3, mean±SEM, two-sided paired t test).

(G) Western blots analysis showing PBRM1 re-expression in 1BR3 parental cells with empty vector (1BR3 Parental Empty) and PBRM1 knockout clone 5 (KO5) carrying an inducible PBRM1 expression construct without or with doxycycline treatment (PBRM1 KO5 rescue -/+ DOX).

(H) Representative images of PBRM1 re-expression of cells in (G). Cells were stained with DAPI and an antibody against PBRM1.

(I) Representative FACS profiles of 1BR3 parental cells with empty vector (1BR3 Parental Empty) with or without DOX induction, untreated (UT) or 24h or 48h post-irradiation.

(J) Quantification of cell cycle phases FACS data of cells in (I) with G1% and G2%. (n=3, mean±SEM).

(K) Quantification of H3S10ph positive (pH3+) G2 cells in 1BR3 parental cells (1BR3 Parental Empty) and PBRM1 KO cells -/+ re-expression of PBRM1 (PBRM1 KO5 rescue -/+ DOX) 48h post-irradiation. (n=3, mean±SEM, two-sided paired t test).

(L) Representative FACS profiles of cells in (K) stained with an antibody against H3S10ph (pH3) and propidium iodide.
