# Supplementary material for: PBAF loss leads to DNA damage-induced inflammatory signaling through defective G2/M checkpoint maintenance
Source: Genes Dev. 2022 Jul 1;36(13-14):790–806. doi: 10.1101/gad.349249.121 (PMC9480851; doi:10.1101/gad.349249.121)
Supplement: Supplemental Material [file supp_gad.349249.121_Supplemental_Figure_S3.pdf]

Feng_Supplemental FigureS3

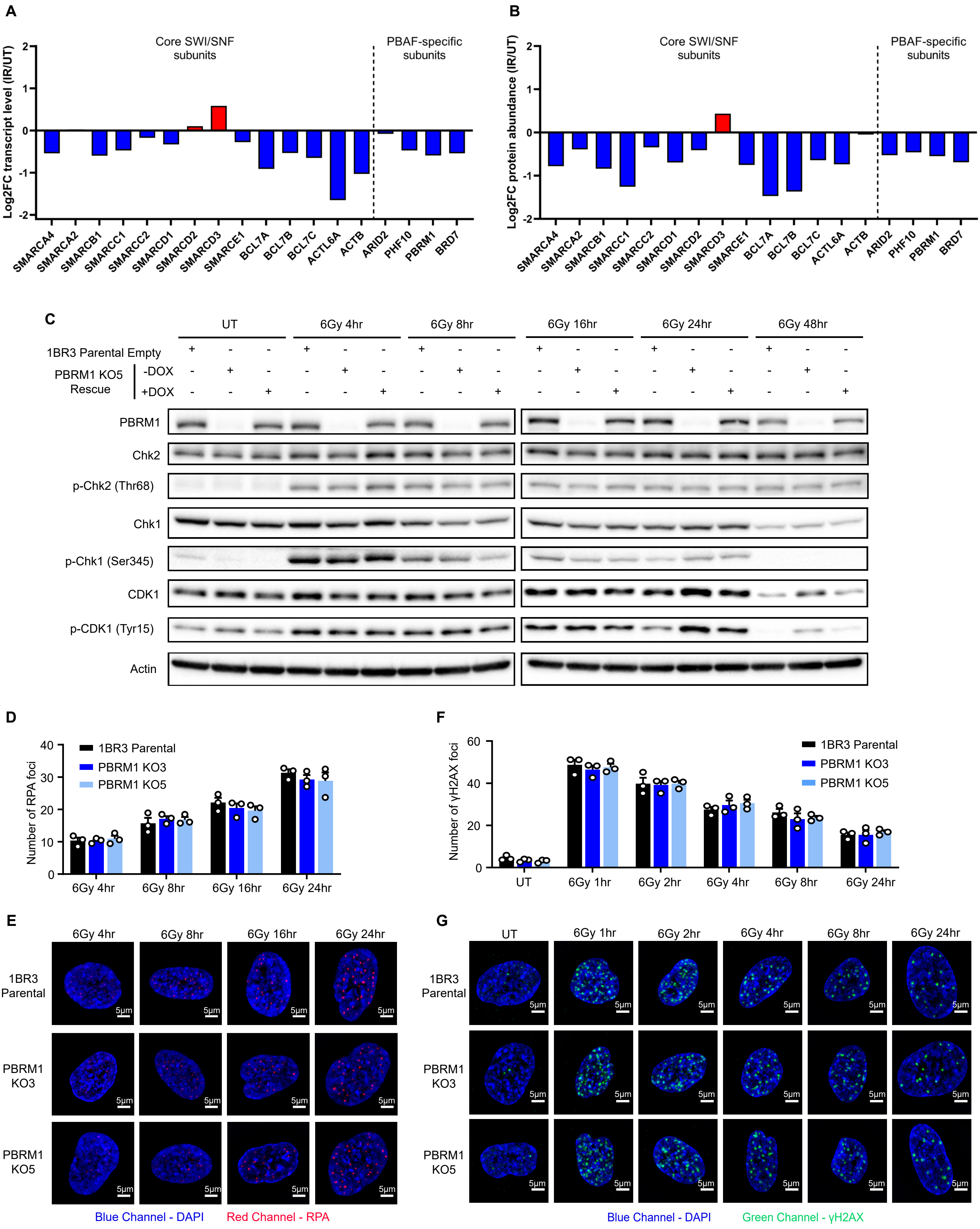

Figure S3. PBRM1 deficiency shows no significant DNA repair defect. Related to Figure 2.

(A) Log2 fold change (Log2FC) in transcript levels of SWI/SNF complex subunits in 1BR3 parental cells 48h after 6Gy irradiation (IR), compared to untreated cells (UT), measured using RNA-seq.

(B) Log2 fold change (Log2FC) of protein abundances of SWI/SNF complex subunits in 1BR3 parental cells 48h after 6Gy irradiation (IR), compared to untreated cells (UT), measured using mass spectrometry data.

(C) Western blot analysis of DNA damage checkpoint response proteins in untreated (UT) or irradiated 1BR3 parental cells with empty vector (1BR3 Parental Empty) and PBRM1 KO cells -/+ re-expression of PBRM1 (PBRM1 KO5 rescue -/+ DOX).

(D) Quantification of RPA foci number per S/G2 nucleus in 1BR3 parental and PBRM1 KO (KO3/5) cells following irradiation. (n=3, mean±SEM).

(E) Representative immunofluorescence images of RPA foci analysis of cells in (A) with an antibody against RPA (red) in cells stained with DAPI (blue).

(F) Quantification of γH2AX foci per nucleus in 1BR3 parental and PBRM1 KO (KO3/5) cells following irradiation. (n=3, mean±SEM).

(G) Representative immunofluorescence images of γH2AX foci analysis of cells in (C) with an antibody against γH2AX (green) in cells stained
