# Supplementary material for: PBAF loss leads to DNA damage-induced inflammatory signaling through defective G2/M checkpoint maintenance
Source: Genes Dev. 2022 Jul 1;36(13-14):790–806. doi: 10.1101/gad.349249.121 (PMC9480851; doi:10.1101/gad.349249.121)
Supplement: Supplemental Material [file supp_gad.349249.121_Supplemental_Figure_S4.pdf]

Feng_Supplemental Figure S4

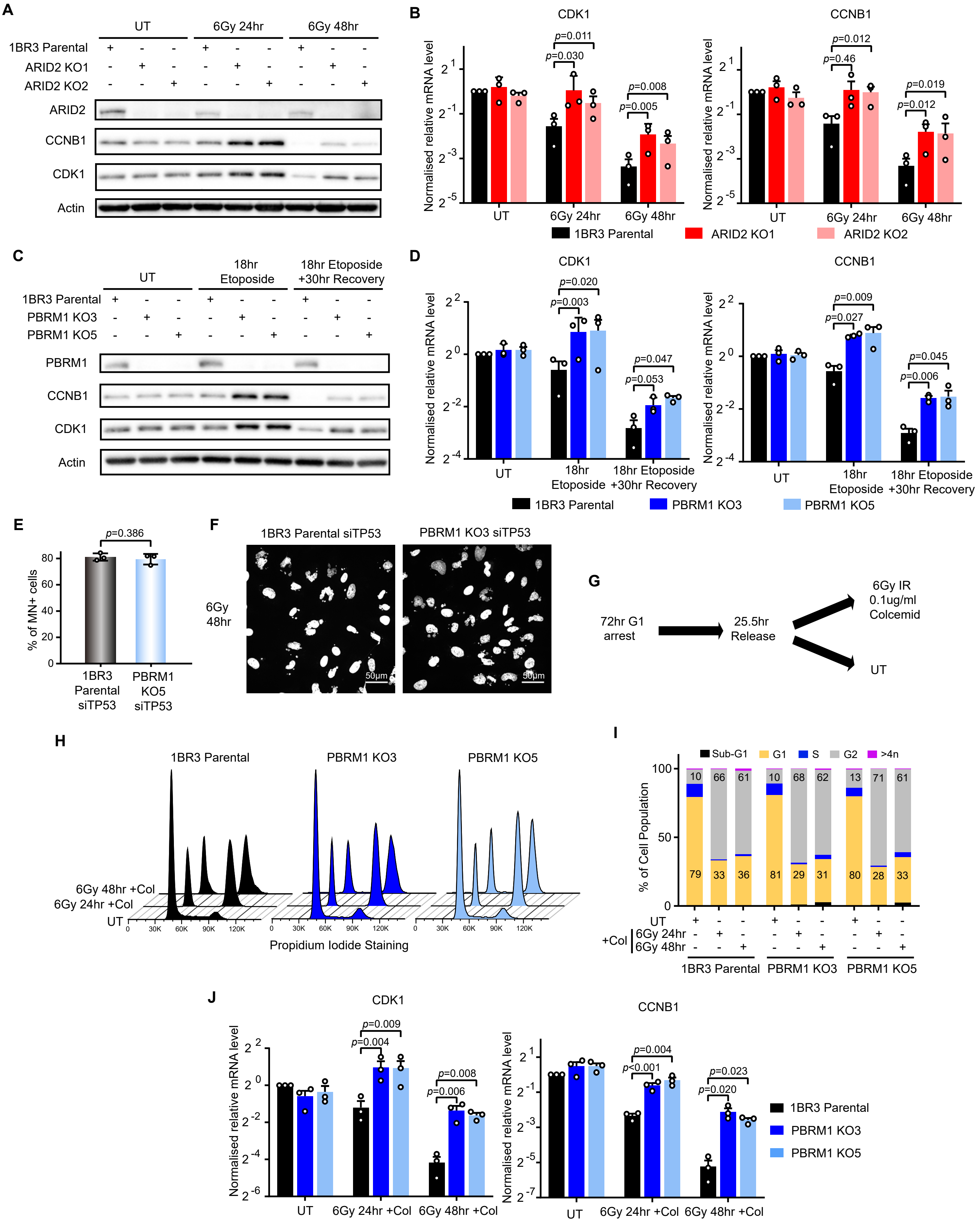

Figure S4. The contribution of PBRM1 to G2/M checkpoint responses is p53-dependent. Related to Figure 2.

(A) Western blot analysis of Cyclin B1 (CCNB1) and CDK1 in untreated (UT) or irradiated 1BR3 parental and ARID2 KO (KO1/2) cells.

(B) RT-qPCR analysis of CDK1 or Cyclin B1 (CCNB1) expression of cells in (A). (n=3, mean±SEM, two-sided paired t test).

(C) Western blot analysis of Cyclin B1 (CCNB1) and CDK1 in untreated (UT) or etoposide treated (18h 500nM etoposide treatment with or without 30h recovery)1BR3 parental and PBRM1 KO (KO3/5) cells.

(D) RT-qPCR analysis of CDK1 or Cyclin B1 (CCNB1) expression of cells in (C). (n=3, mean±SEM, two-sided paired t test).

(E) Quantification of cells with micronuclei in untreated or irradiated 1BR3 parental and PBRM1 KO5 cells treated with siRNA depletion of TP53 (siTP53) or non-targeting control (siCON).

(F) Representative images of DAPI stained irradiated cells in (E).

(G) Flow chart showing treatment profile of G1 confluence arrest, release, and irradiation with colcemid treatment.

(H) Representative FACS profiles of 1BR3 parental and PBRM1 KO (KO3/5) cells following G1 arrest and release, untreated (UT) or irradiated with colcemid treatment.

(I) Quantification of cell cycle phases FACS data of cells in (H) with G1% and G2%. (n=1).

(J) RT-qPCR analysis of CDK1 and Cyclin B1 (CCNB1) expression of cells in (H). (n=3, mean±SEM, two-sided paired t test).
