# Supplementary material for: PBAF loss leads to DNA damage-induced inflammatory signaling through defective G2/M checkpoint maintenance
Source: Genes Dev. 2022 Jul 1;36(13-14):790–806. doi: 10.1101/gad.349249.121 (PMC9480851; doi:10.1101/gad.349249.121)
Supplement: Supplemental Material [file supp_gad.349249.121_Supplemental_Figure_S5.pdf]

Feng_Supplemental Figure S5

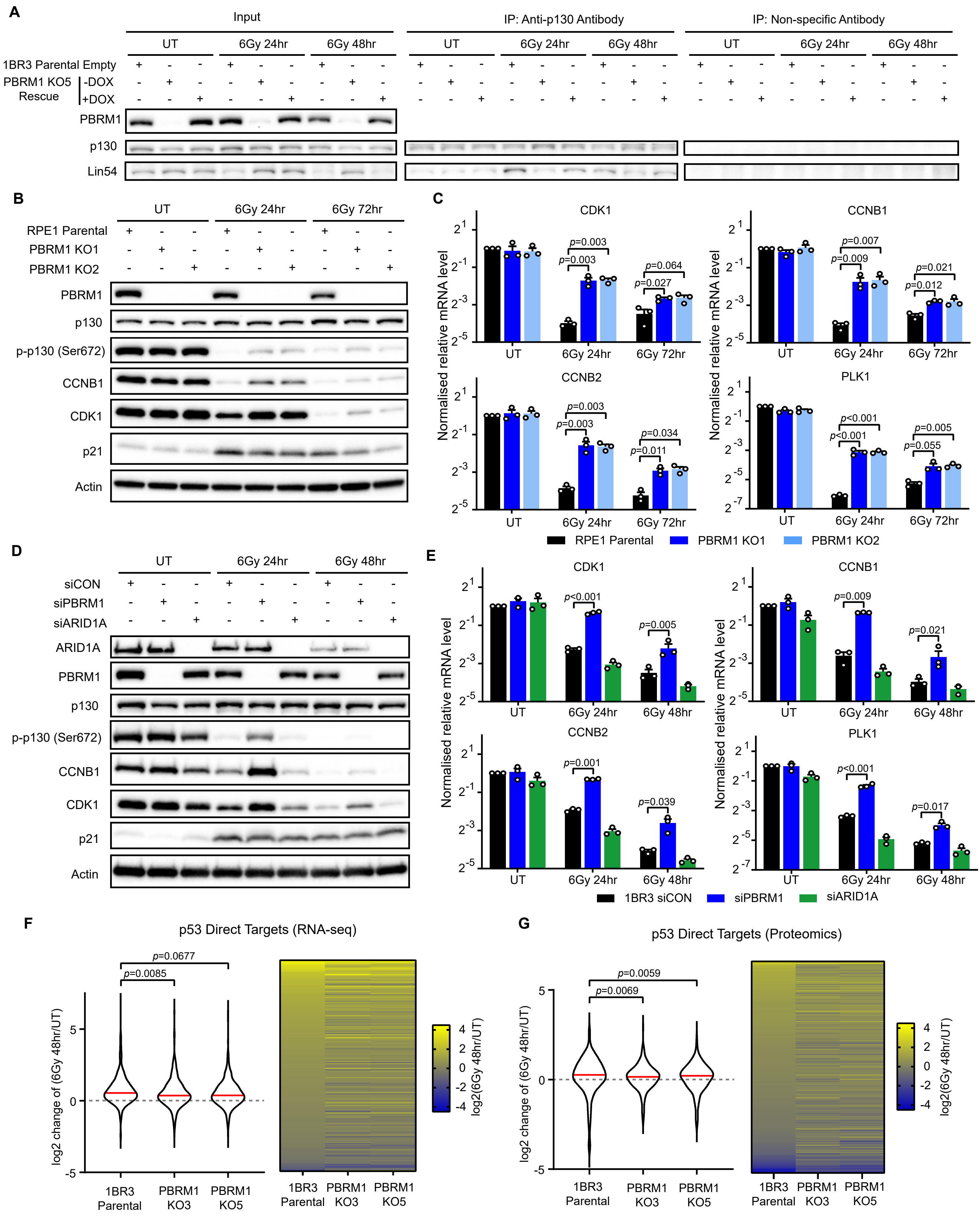

Figure S5. PBRM1 mediates p53-dependent CDK1 and Cyclin B1 repression via the DREAM pathway. Related to Figure 3.

(A) Western blot analysis of p130 and Lin54 with co-immunoprecipitation using a non-specific antibody (negative control) or anti-p130 antibody in untreated (UT) or irradiated 1BR3 parental cells (1BR3 Parental Empty) and PBRM1 KO cells -/+ re-expression of PBRM1 (PBRM1 KO5 rescue -/+ DOX).

(B) Western blot analysis of RPE1 parental and PBRM1 KO (KO1/2) cells, untreated (UT) or 24h or 72h post-irradiation.

(C) RT-qPCR analysis of CDK1, Cyclin B1 and B2 (CCNB1 and CCNB2), and PLK1 expression of cells in (B). (n=3, mean±SEM, two-sided paired t test).

(D) Western blot analysis of 1BR3 parental cells with siRNA depletion of PBRM1 (siPBRM1) or ARID1A (siARID1A) or non-targeting control (siCON), untreated (UT) or 24h or 48h post-irradiation.

(E) RT-qPCR analysis of CDK1, Cyclin B1 and B2 (CCNB1 and CCNB2), and PLK1 expression of cells in (D). (n=3, mean±SEM, two-sided paired t test).

(F, G) Violin plot and heat map showing changes in p53 direct target transcript levels from RNA-seq data (F) or p53 direct target protein levels from mass spectrometry data (G) in 1BR3 parental or PBRM1 KO (KO3/5) cells between untreated (UT) and 48h post-irradiation. Data are plotted as the log2 ratio of values from irradiated versus untreated samples (log2(6Gy 48hr/UT)). The red line in the violin plot indicates the median. (two-tailed Wilcoxon rank sum test with continuity correction).
