# Supplementary material for: PBAF loss leads to DNA damage-induced inflammatory signaling through defective G2/M checkpoint maintenance
Source: Genes Dev. 2022 Jul 1;36(13-14):790–806. doi: 10.1101/gad.349249.121 (PMC9480851; doi:10.1101/gad.349249.121)
Supplement: Supplemental Material [file supp_gad.349249.121_Supplemental_Figure_S6.pdf]

Feng_Supplemental Figure S9

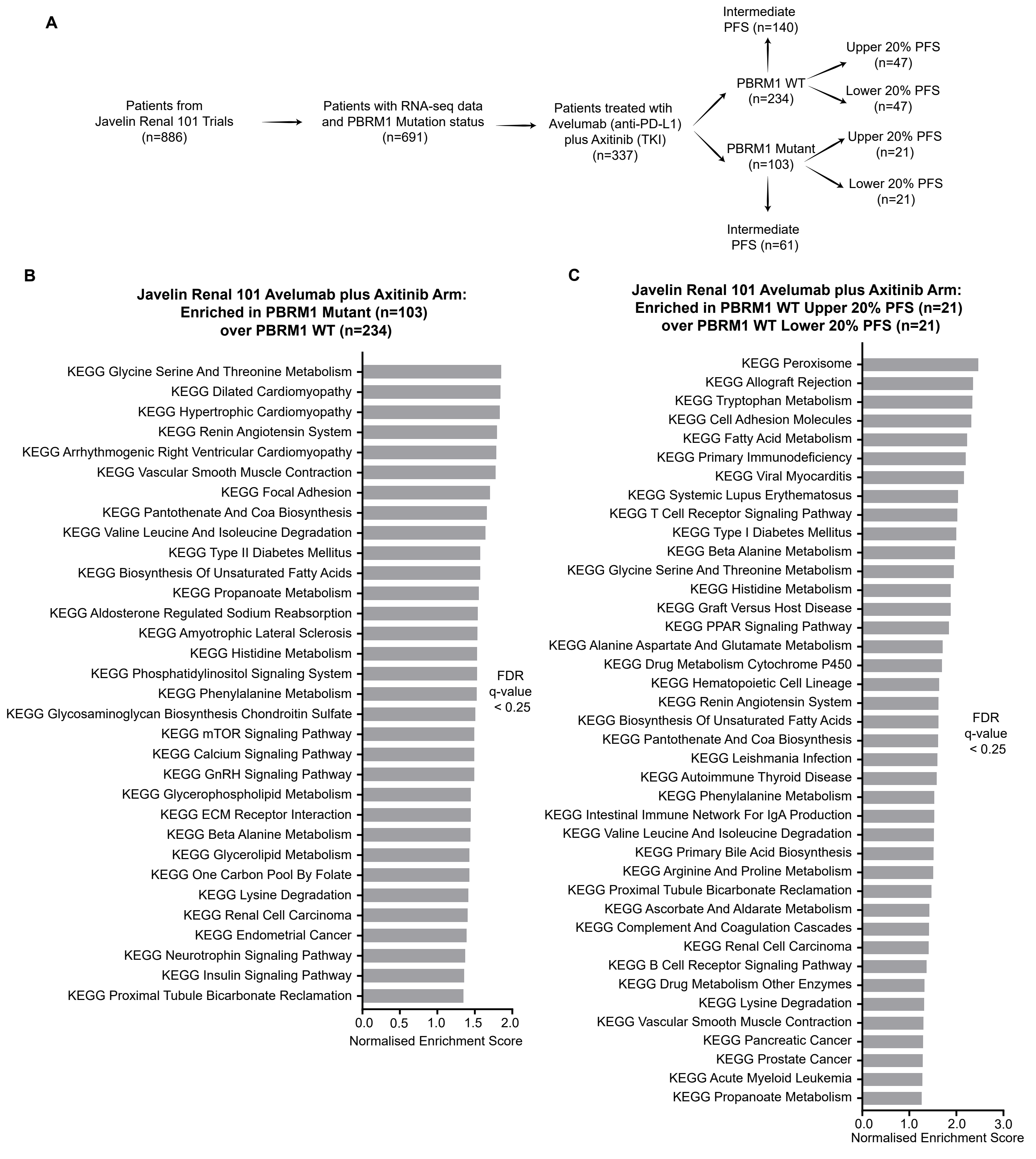

Figure S9. GSEA analysis in different patient groups from the Javelin Renal 101 clinical trial. Related to Figure 6.

(A) Flow chart of patient stratification for analysis from the Javelin Renal 101 trial data.

(B) Significantly enriched KEGG gene sets (False Discovery Rate (FDR) q-value <0.25) in the PBRM1 mutant over the PBRM1 wild-type (WT) patients treated with Avelumab plus Axitinib as stratified in (A).

(C) Significantly enriched KEGG gene sets (False Discovery Rate (FDR) q-value <0.25) in the PBRM1 WT patients treated with Avelumab plus Axitinib with upper 20% progression free survival (PFS) over and lower 20% PFS as stratified in (A).
