# Supplementary material for: PBAF loss leads to DNA damage-induced inflammatory signaling through defective G2/M checkpoint maintenance
Source: Genes Dev. 2022 Jul 1;36(13-14):790–806. doi: 10.1101/gad.349249.121 (PMC9480851; doi:10.1101/gad.349249.121)
Supplement: Supplemental Material [file supp_gad.349249.121_Supplemental_Figure_S7.pdf]

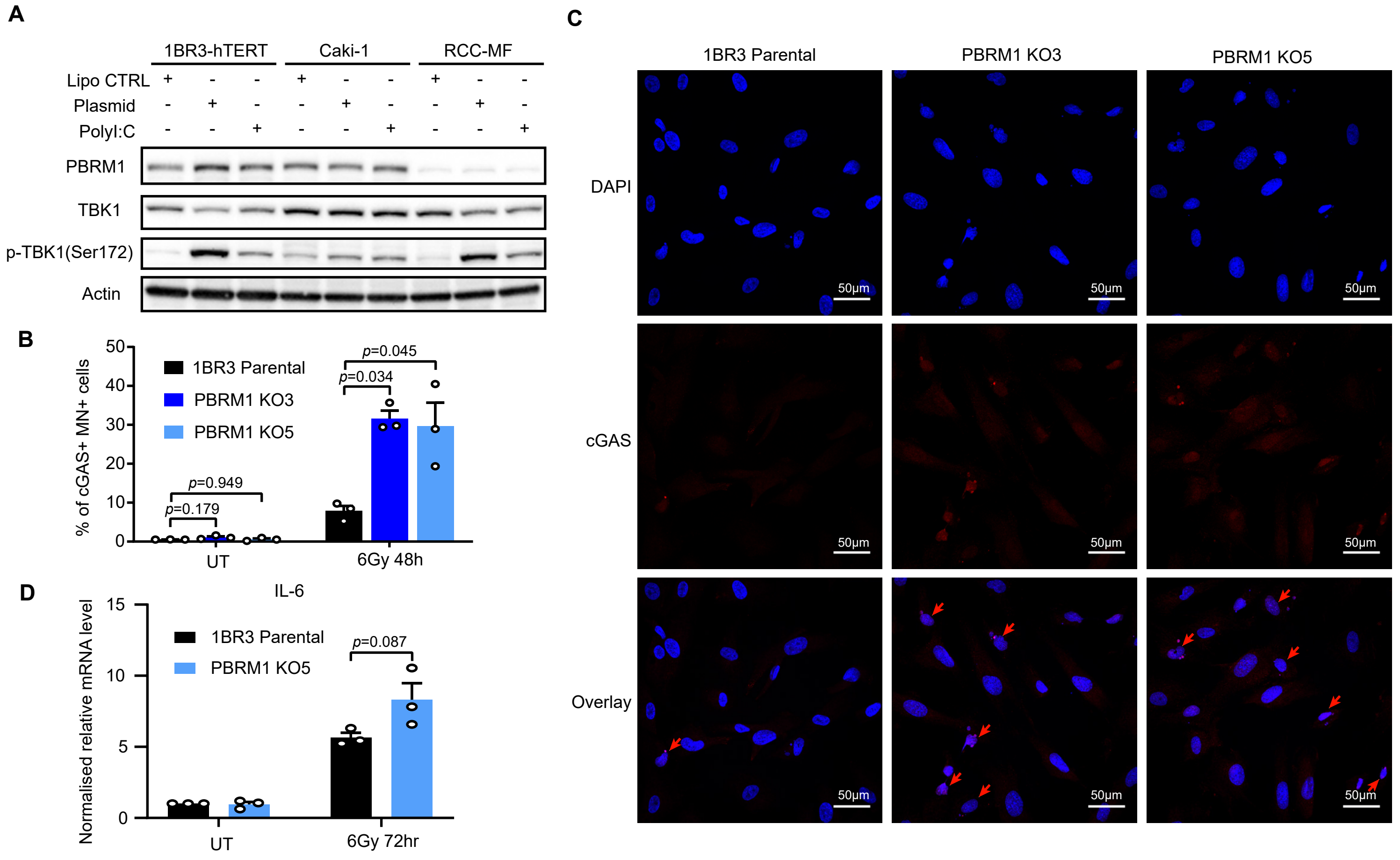
A
1BR3-hTERT
Caki-1
RCC-MF
Lipo CTRL
Plasmid
PolyI:C
PBRM1
TBK1
p-TBK1(Ser172)
Actin
B
% of cGAS+ MN+ cells
1BR3 Parental
PBRM1 KO3
PBRM1 KO5
p=0.045
p=0.034
p=0.949
p=0.179
UT
6Gy 48h
D
IL-6
Normalised relative mRNA level
1BR3 Parental
PBRM1 KO5
p=0.087
UT
6Gy 72hr
C
1BR3 Parental
PBRM1 KO3
PBRM1 KO5
DAPI
cGAS
Overlay
50μm

Figure S7. Analysis of PBRM1 deficiency and DNA damage induced inflammatory genes. Related to Figure 5.

(A) Western blot analysis of the 1BR3-hTERT, Caki-1 and RCC-MF cell lines following mock transfection (Lipo CTRL) or transfection with a DNA plasmid or polyI:C (dsRNA mimic) to monitor nucleic acid sensing signalling responses using phosphorylated TBK1 (p-TBK1) as a readout of activation.

(B) Quantification of cells with cGAS positive micronuclei in untreated (UT) or irradiated 1BR3 parental and PBRM1 KO (KO3/5) cells. (n=3, mean±SEM, two-sided paired t test).

(C) Representative images of cells in (B). Cells were stained with DAPI and an antibody against cGAS. cGAS positive micronuclei are indicated with arrows.

(D) RT-qPCR of IL-6 in untreated (UT) or irradiated 1BR3 parental and PBRM1 KO (KO3/5) cells. (n=3, mean±SEM, two-sided paired t test).
