# Supplementary material for: PBAF loss leads to DNA damage-induced inflammatory signaling through defective G2/M checkpoint maintenance
Source: Genes Dev. 2022 Jul 1;36(13-14):790–806. doi: 10.1101/gad.349249.121 (PMC9480851; doi:10.1101/gad.349249.121)
Supplement: Supplemental Material [file supp_gad.349249.121_Supplemental_Figure_S8.pdf]

Feng_Supplemental Figure S8

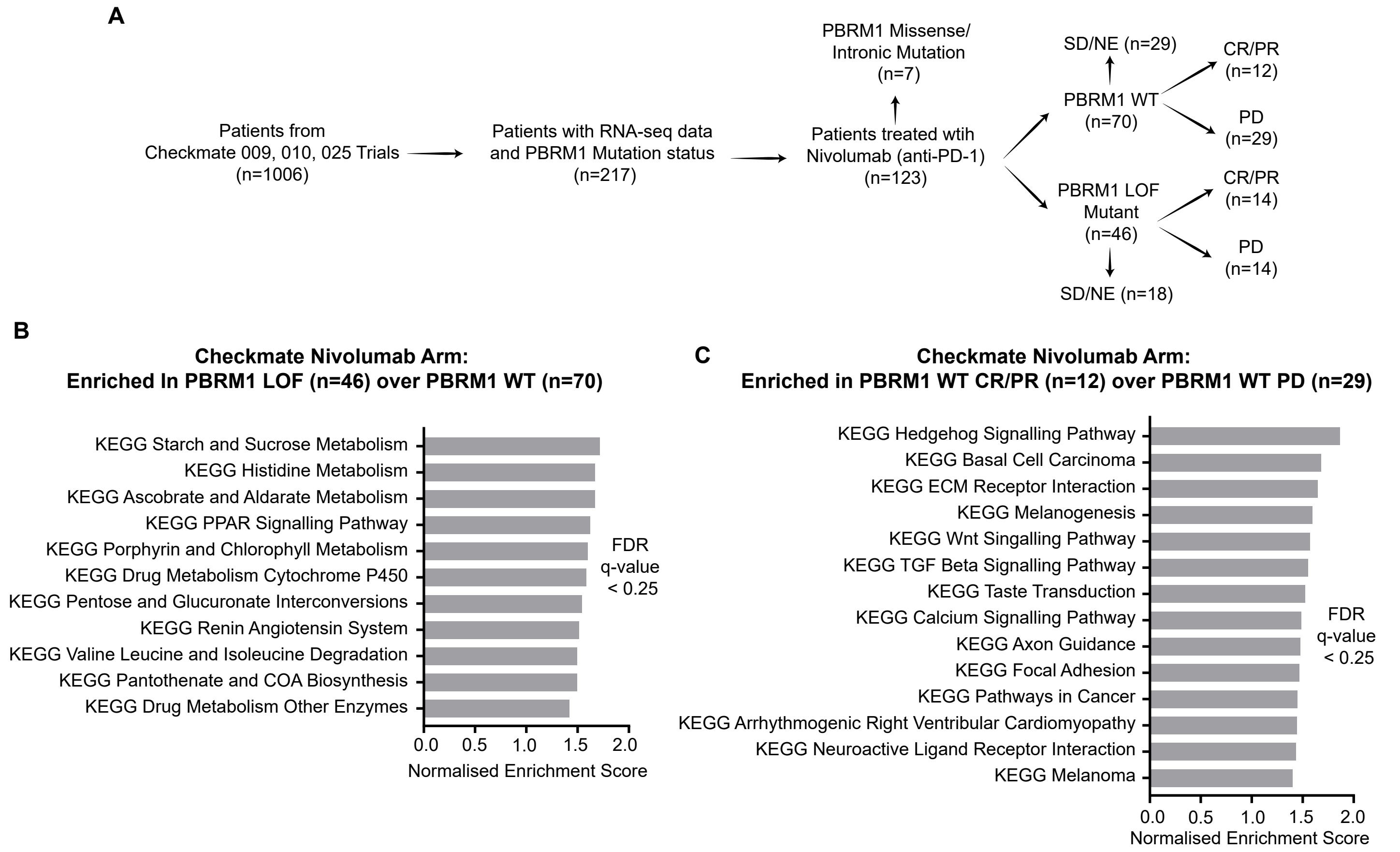

Figure S8. GSEA analysis in different patient groups from the Checkmate clinical trials. Related to Figure 6.

(A) Flow chart of patient stratification for analysis from the Checkmate clinical trial data.

(B) Significantly enriched KEGG gene sets (False Discovery Rate (FDR) q-value <0.25) in the PBRM1 loss-of-function (LOF) mutant over the PBRM1 wild-type (WT) patients treated with Nivolumab as stratified in (A).

(C) Significantly enriched KEGG gene sets (False Discovery Rate (FDR) q-value <0.25) in the PBRM1 WT patients treated with Nivolumab with complete or partial response (CR/PR) over with progressive disease (PD) as stratified in (A).
