# Supplementary material for: PBAF loss leads to DNA damage-induced inflammatory signaling through defective G2/M checkpoint maintenance
Source: Genes Dev. 2022 Jul 1;36(13-14):790–806. doi: 10.1101/gad.349249.121 (PMC9480851; doi:10.1101/gad.349249.121)
Supplement: Supplemental Material [file supp_gad.349249.121_Supplemental_Tabel_S1.pdf]

Cell line	Genotype	Source	PBRM1 status
1BR3-hTERT	Parental	Prof. Penny Jeggo	WT (wild type)
	PBRM1 KO3	This study	KO
	PBRM1 KO5	This study	KO
	PBRM1 KO5 Rescue	This study	KO + expression construct
	ARID2 KO1	This study	WT
	ARID2 KO2	This study	WT
hTERT-RPE1	Parental	ATCC	WT
	PBRM1 KO1	This study	KO
	PBRM1 KO2	This study	KO
Caki-1	Parental	ATCC	WT
RCC-MF	Parental	CLS	Null (p.N528fs - frameshift truncating mutation)
	PBRM1 rescue	This study	Null mutant + expression construct

Table S1. Cell line information. Related to Materials and Methods.
