# Supplementary material for: PBAF loss leads to DNA damage-induced inflammatory signaling through defective G2/M checkpoint maintenance
Source: Genes Dev. 2022 Jul 1;36(13-14):790–806. doi: 10.1101/gad.349249.121 (PMC9480851; doi:10.1101/gad.349249.121)
Supplement: Supplemental Material [file supp_gad.349249.121_Supplemental_Table_S2.pdf]

Primer	Sequence (5' – 3')	Application
hPBRM1_sgRNA2_FW	CACCGTCAGCGGGGACTTTGATGAT	CRISPR sgRNA
hPBRM1_sgRNA2_RV	AAACATCATCAAAGTCCCCGCTGAC	CRISPR sgRNA
hARID2_sgRNA2_FW	CACCGCCTCCGGACGAGCGGAGAAA	CRISPR sgRNA
hARID2_sgRNA2_RV	AAACTTTCTCCGCTCGTCCGGAGGC	CRISPR sgRNA
hPBRM1_gDNA_FW	TATCCTCCTAAGTGAGCCAGG	CRISPR KO Genotyping
hPBRM1_gDNA_RV	TTACGATGCAAGGCATCTCC	CRISPR KO Genotyping
hARID2_gDNA_FW	TAATGGCAAACTCGACGGGG	CRISPR KO Genotyping
hARID2_gDNA_RV	TTCGCGAATCCGCCTAAAGT	CRISPR KO Genotyping
GAPDH_RT-qPCR_FW	ATTCCACCCATGGCAAATTC	RT-qPCR
GAPDH_RT-qPCR_RV	TCTCGCTCCTGGAAGATGGT	RT-qPCR
CDK1_RT-qPCR_FW	GCGGAATAATAAGCCGGGAT	RT-qPCR
CDK1_RT-qPCR_RV	CAACTCCATAGGTACCTTCTCCA	RT-qPCR
CCNB1_RT-qPCR_FW	GTTATGCAGCACCTG	RT-qPCR
CCNB1_RT-qPCR_RV	CTTGGCTAAATCTTGAACT	RT-qPCR
CCNB2_RT-qPCR_FW	GCGTTGGCATTATGGATCG	RT-qPCR
CCNB2_RT-qPCR_RV	TCTTCCGGGAAACTGGCTG	RT-qPCR
PLK1_RT-qPCR_FW	GCACAGTGTCAATGCCTCCAAG	RT-qPCR
PLK1_RT-qPCR_RV	GCCGTACTTGTCCGAATAGTCC	RT-qPCR
IL6_RT-qPCR_FW	GGTACATCCTCGACGGCATC	RT-qPCR
IL6_RT-qPCR_RV	GGTTGTTTTCTGCCAGTGCC	RT-qPCR
β-IFN_RT-qPCR_FW	AAACTCATGAGCAGTCTGCA	RT-qPCR
β-IFN_RT-qPCR_FW	AGGAGATCTTCAGTTTCGGAGG	RT-qPCR
CCL5_RT-qPCR_FW2	TCTGCGCTCCTGCATCTG	RT-qPCR
CCL5_RT-qPCR_RV2	GGGCAATGTAGGCAAAGCA	RT-qPCR
CXCL10_RT-qPCR_FW	TGCCATTCTGATTTGCTGCC	RT-qPCR
CXCL10_RT-qPCR_RV	TGCAGGTACAGCGTACAGTT	RT-qPCR
p21_RT-qPCR_FW	AGGTGGACCTGGAGACTCTCAG	RT-qPCR
p21_RT-qPCR_RV	TCCTCTTGGAGAAGATCAGCCG	RT-qPCR
CDK1-CDE_ChIP_qPCR_FW	TGCTTTGAAAGTCTACGGGCT	ChIP-qPCR
CDK1-CDE_ChIP_qPCR_RV	CGTCGCTCTCCGCTCAATTT	ChIP-qPCR
CCNB1-CDE_ChIP_qPCR_FW	CCACGAACAGGCCAATAAGG	ChIP-qPCR
CCNB1-CDE_ChIP_qPCR_RV	GAAACCAACAGCCGTTCCG	ChIP-qPCR
P21-RE1_ChIP_qPCR_FW	AGCAGGCTGTGGCTCTGATT	ChIP-qPCR
P21-RE1_ChIP_qPCR_RV	CAAAATAGCCACCAGCCTCTTCT	ChIP-qPCR
TRIPZ_Empty_Frag_FW	AATTCAAGGGGCTACTTTAGGAGCAATTATCTTGTTT ACTAAAACTGAAT	Empty-TRPIZ-neo Cloning
TRIPZ_Empty_Frag_RV	TCAGTTTTAGTAAACAAGATAATTGCTCCTAAAGTAG CCCCTTG	Empty-TRPIZ-neo Cloning

Table S2. DNA oligonucleotide information. Related to Materials and Methods.
