# Supplementary material for: PBAF loss leads to DNA damage-induced inflammatory signaling through defective G2/M checkpoint maintenance
Source: Genes Dev. 2022 Jul 1;36(13-14):790–806. doi: 10.1101/gad.349249.121 (PMC9480851; doi:10.1101/gad.349249.121)
Supplement: Supplemental Material [file supp_gad.349249.121_Supplemental_Table_S3.pdf]

Antibody/Target	Source	Catalogue	Application&Dilution
Mouse Monoclonal anti-Actin/HRP	Sigma	A3854	WB 1:40000
Mouse Monoclonal anti-ARID2	Abcam	ab51019	WB 1:250
Mouse Monoclonal anti-CDK1	BD Biosciences	610038	WB 1:1000
Mouse Monoclonal anti-Phos-CDK1 (Y15)	BD Biosciences	612307	WB 1:1000
Rabbit Monoclonal anti-cGAS	Cell Signalling Technology	15102	IF 1:300
Rabbit Monoclonal anti-Chk1	Cell Signalling Technology	2360	WB 1:1000
Rabbit Monoclonal anti-Phos-Chk1 (S345)	Cell Signalling Technology	2341	WB 1:500
Rabbit Monoclonal anti-Chk2	Cell Signalling Technology	6334	WB 1:1000
Rabbit Monoclonal anti-Phos-Chk2 (T68)	Cell Signalling Technology	2197	WB 1:500
Mouse Monoclonal anti-Cyclin B1	Santa Cruz	sc-245	WB 1:200
Rabbit Monoclonal anti-Phos-H3 (Ser10)	Abcam	ab14955	FACS 1:500
Rabbit Polyclonal anti-Lin54	Bethyl	A303-799A	WB: 1:1000
Mouse Monoclonal anti-P130	Santa Cruz	sc-53641	ChIP 1ug per 1 x 10^7 cells<br>CoIP 2.5ug per 2500µg Protein
Mouse Monoclonal anti-RB2 (P130)	BD Biosciences	610262	WB 1:200
Rabbit Monoclonal anti-Phos-P130 (S672)	Abcam	ab76255	WB 1:500
Rabbit Monoclonal anti-P21	Cell Signalling Technology	2947	WB 1:2000
Mouse Monoclonal anti-p53	Santa Cruz	sc-126	ChIP 1ug per 1 x 10^7 cells
Rabbit polyclonal anti-PBRM1	Novus	NBP2-55731	WB 1:500; IF 1:100
Rabbit Polyclonal anti-Rad51	Santa Cruz	sc-8349	IF 1:250
Rat Monoclonal anti-RPA2	LSBio	LS-C38952	IF 1:800
Mouse Monoclonal anti-α-tubulin	Abcam	ab7291	WB 1:10000
Mouse Monoclonal anti-γH2AX(Ser139)	Millipore	05-636	IF 1:500
Goat anti-Mouse IgG/Alexa Fluor 555	Thermo Fisher Scientific	A-21422	IF 1:500
Donkey anti-Rat IgG/Alexa Fluor 488	Thermo Fisher Scientific	A-21208	IF 1:500
Goat Anti-Mouse IgG/Alexa Fluor 647	Thermo Fisher Scientific	A-21235	FACS 1:500
Sheep Anti-rabbit IgG/Cy3	Sigma-Aldrich	C2306	IF 1:500
Goat Anti-Mouse IgG/FITC	Sigma-Aldrich	F0257	IF 1:500
Goat Anti-Rabbit Immunoglobulins/HRP	Agilent (Dako)	P044801-2	WB 1:5000
Rabbit Anti-Mouse Immunoglobulins/HRP	Agilent (Dako)	P026002-2	WB 1:5000
Mouse polyclonal non-specific	Millipore	12-371	ChIP 1ug per 1 x 10^7 cells<br>CoIP 2.5ug per 2500µg Protein

Table S3. Antibody Information. Related to Materials and Methods.
